# Cholesterol accumulation impairs HIF-1α-dependent immunometabolic reprogramming of LPS-stimulated macrophages by upregulating the NRF2 pathway

**DOI:** 10.1038/s41598-024-61493-6

**Published:** 2024-05-15

**Authors:** Kenneth K. Y. Ting, Pei Yu, Riley Dow, Hisham Ibrahim, Saraf Karim, Chanele K. Polenz, Daniel A. Winer, Minna Woo, Jenny Jongstra-Bilen, Myron I. Cybulsky

**Affiliations:** 1 Department of Immunology, University of Toronto, Toronto, ON M5S 1A8 Canada; 2 Toronto General Hospital Research Institute, University Health Network, PMCRT 3-306, 101 College Street, TMDT, Toronto, ON M5G 1L7 Canada; 3 Department of Laboratory Medicine and Pathobiology, University of Toronto, Toronto, ON M5S 1A8 Canada; 4 Division of Cellular & Molecular Biology, Diabetes Research Group, Toronto General Hospital Research Institute, University Health Network, Toronto, ON M5G 1L7 Canada; 5 Division of Endocrinology and Metabolism, Department of Medicine, University Health Network, University of Toronto, Toronto, ON M5S 1A8 Canada; 6 Banting and Best Diabetes Centre, University of Toronto, Toronto, ON M5G 2C4 Canada; 7 Peter Munk Cardiac Centre, University Health Network, Toronto, ON M5G 2N2 Canada

**Keywords:** Monocytes and macrophages, Chronic inflammation, Atherosclerosis, Pattern recognition receptors

## Abstract

Lipid accumulation in macrophages (Mφs) is a hallmark of atherosclerosis. Yet, how lipid loading modulates Mφ inflammatory responses remains unclear. We endeavored to gain mechanistic insights into how pre-loading with free cholesterol modulates Mφ metabolism upon LPS-induced TLR4 signaling. We found that activities of prolyl hydroxylases (PHDs) and factor inhibiting HIF (FIH) are higher in cholesterol loaded Mφs post-LPS stimulation, resulting in impaired HIF-1α stability, transactivation capacity and glycolysis. In RAW264.7 cells expressing mutated HIF-1α proteins resistant to PHDs and FIH activities, cholesterol loading failed to suppress HIF-1α function. Cholesterol accumulation induced oxidative stress that enhanced NRF2 protein stability and triggered a NRF2-mediated antioxidative response prior to and in conjunction with LPS stimulation. LPS stimulation increased NRF2 mRNA and protein expression, but it did not enhance NRF2 protein stability further. NRF2 deficiency in Mφs alleviated the inhibitory effects of cholesterol loading on HIF-1α function. Mutated KEAP1 proteins defective in redox sensing expressed in RAW264.7 cells partially reversed the effects of cholesterol loading on NRF2 activation. Collectively, we showed that cholesterol accumulation in Mφs induces oxidative stress and NRF2 stabilization, which when combined with LPS-induced NRF2 expression leads to enhanced NRF2-mediated transcription that ultimately impairs HIF-1α-dependent glycolytic and inflammatory responses.

## Introduction

Atherosclerosis is a chronic inflammatory disease that is dependent upon responses orchestrated by immune cells located within the aortic intima. Macrophages (Mφs) that reside in the aortic intima (Mac^AIR^) are found in regions predisposed to atherosclerotic lesion formation under homeostatic conditions^1^. During hypercholesterolemia, these cells rapidly accumulate intracellular lipid, which leads to the formation of intimal foam cells and nascent atherosclerotic lesions^2^. Subsequently, a chronic inflammatory response enhances the recruitment of monocytes circulating in the arterial blood to the intima^2,3^. These cells are retained locally, differentiate into Mφs and also accumulate intracellular lipid, becoming foam cells that contribute to lesion progression^3^.

While foam cells play an indisputable role in atherogenesis, it remains unclear whether foam cells drive inflammation during atherogenesis and how lipid loading modulates Mφ inflammatory responses. Previous reports have shown that the intrinsic accumulation of lipoprotein-derived cholesterol in Mφs is sufficient to drive inflammation in a NLRP3-dependent manner^4^. Other studies have demonstrated that cholesterol loading of Mφs impaired their ability to induce an effective inflammatory response by LXR-dependent and -independent mechanisms^5–8^. More recently, new findings from both mouse and human atherosclerotic lesions have shown that foamy Mφs were less inflammatory than non-foamy ones^9,10^, thus strengthening the notion that lipid loading is not an intrinsically inflammatory process.

The immunometabolism field has comprehensively demonstrated that the induction of glycolysis is critical for orchestrating myeloid cell inflammatory responses^11^. Specifically, appropriately stimulated myeloid cells undergo phases of glycolytic reprogramming to fuel the function of their inflammatory machinery, such as the early phase of glycolytic influx mediated by AKT^12^, and the late phase glycolysis that is mediated by the stabilization of hypoxia inducing factor-1α (HIF-1α), which is critical for the transcription of glycolysis and inflammatory genes, such as IL-1β^13^.

Hypoxia-inducible factor (HIF)-1 is a heterodimeric transcription factor that is composed of an α and β subunit, in which the α subunit is unstable and oxygen-sensitive, while the β subunit is constitutively expressed and oxygen-insensitive^14–16^. Therefore, the transcriptional activity of HIF-1 is critically dependent on the stability of HIF-1α. In the classical model of HIF-1α degradation, key conserved proline residues of HIF-1α proteins are hydroxylated by prolyl hydroxylases (PHDs), which facilitate the binding of von Hippel-Lindau (VHL) ubiquitin ligase complex to HIF-1α and target it for proteasomal degradation^17–20^. Apart from PHDs, HIF-1α proteins are also hydroxylated by factor inhibiting HIF (FIH) at a key conserved asparagine residue, which blocks the recruitment of HIF-1α coactivators, such as p300/CBP, thereby impairing the transactivation capacity of HIF-1^21^. Although the roles of PHDs and FIH in regulating HIF-1α stabilization and transactivation is well-established in the context of hypoxia, how they regulate HIF-1α function in inflammatory macrophages under normoxic conditions is not well defined.

Our group has previously shown that oxidized low-density lipoprotein (oxLDL) accumulation in Mφs upregulates nuclear factor-erythroid factor 2-related factor 2 (NRF2)-dependent antioxidative response and suppresses HIF-1α-dependent glycolysis and inflammation in response to LPS^22,23^. However, it remains unknown if loading of free cholesterol will promote a similar mechanism. In addition, it is not well understood how lipid loading of Mφs mechanistically upregulates the NRF2 pathway. Kelch-like erythroid cell-derived protein with CNC homology-associated protein 1 (KEAP1), a highly conserved and cysteine rich protein (27 cysteines in human and 25 in mouse KEAP1), is a substrate adaptor of a E3-ligase complex that constitutively targets NRF2 for proteasomal degradation^24^. The free thiol groups found on the cysteine residues of KEAP1, such as C151, C273 and C288, can readily react with electrophiles and thus function as redox sensors^24^. Upon oxidative stress, electrophiles induce post-translational modifications on these cysteine residues causing conformational changes to KEAP1, escape of NRF2 from degradation and transcriptional activation of antioxidative defense genes^24^.

In this study, we demonstrated that the accumulation of free cholesterol elevated the activity of PHDs and FIH in LPS-stimulated Mφs, thereby impairing HIF-1α stabilization and transactivation capacity, respectively. This shows that the metabolic adaptation of Mφs to loading with cholesterol or oxLDL is similar. We also enhanced our understanding of the mechanism by showing that cholesterol loading alone induced oxidative stress, impaired KEAP1 function through modification of cysteine-151 and stabilized NRF2 protein in Mφs prior to LPS stimulation. The pre-stabilization of NRF2, together with LPS-induced expression of NRF2 mRNA and protein, led to enhanced NRF2-regulated transcription of antioxidant genes and the suppression of HIF-1-dependent glycolytic and inflammatory responses.

## Results

### Cholesterol loading of Mφs impairs LPS-induced HIF-1α stabilization and glycolysis

**Figure 1:**
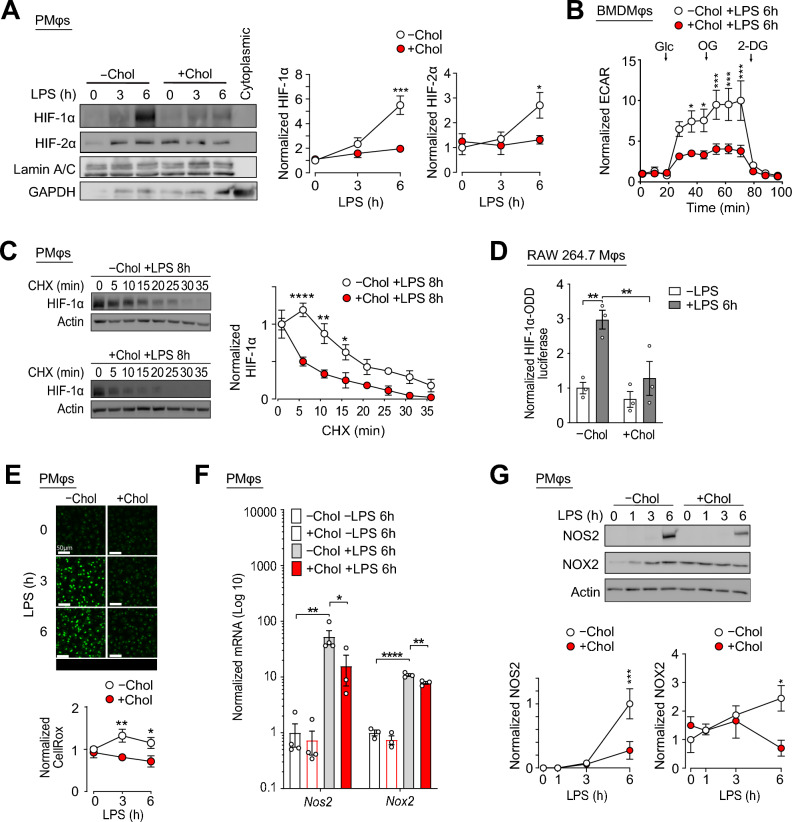
Cholesterol loading of Mφs impairs LPS-induced HIF-1α stabilization. (**A**) Representative immunoblots and quantification of a LPS time course (0, 3 and 6 h) showing nuclear HIF-1α and HIF-2α protein accumulation in PMφs with (+) or without (−) cholesterol (Chol) loading. Data are normalized to lamin A/C and the -Chol 0 h LPS time point (assigned a value of 1, n = 3). (**B**) Glycolysis stress test showing ECAR (normalized to baseline, assigned a value of 1) in BMDMφs with and without cholesterol loading and 6 h after LPS stimulation (n = 4). Arrows indicate injections of glucose (Glc), oligomycin (OG), and 2-deoxyglucose (2-DG). (**C**) Assessment of HIF-1α protein stability. Representative immunoblots and quantification showing HIF-1α protein accumulation in PMφs with and without cholesterol loading and LPS stimulation (8 h) after cycloheximide (CHX) treatment (0–35 min). HIF-1α values are normalized to the corresponding actin and the pre-CHX time point (assigned a value of 1, n = 3). (**D**) Effect of LPS stimulation (6 h) on PHD activity in RAW264.7 cells transfected with luciferase reporter. HIF-1α-ODD firefly luciferase activity is normalized to Renilla luciferase activity to account for transfection efficiency and to values found in control cells (n = 3). (**E**) Representative confocal microscope images and quantification of ROS (CellROX staining, green) in PMφs with and without cholesterol loading and LPS stimulation (0, 3 and 6 h). Values are normalized to the –Chol 0 h LPS time point (assigned a value of 1, n = 9–12, scale bars, 50 µm). (**F**) qPCR analysis of *Nox2* and *Nos2* mRNA in PMφs with and without cholesterol loading and LPS stimulation (0 or 6 h, n = 3–4). The mean ± SEM is plotted in all graphs. (**G**) Immunoblot analysis and quantification of NOX2 and NOS2 protein in PMφs with and without cholesterol loading and LPS stimulation (0–6 h). Data are normalized to the corresponding actin and –Chol value at 6 (NOS2) or 0 (NOX2) h post LPS (assigned a value of 1, n = 3). The mean ± SEM is plotted in all graphs. Significant differences are determined by a two-way ANOVA with Bonferroni correction (*P < 0.05, **P < 0.01, ***P < 0.001, ****P < 0.0001).

HIF-1, a transcription factor that transcribes glycolysis genes^25^, is critical for the induction of glycolysis in LPS-activated Mφs as this metabolic rewiring is important for optimal inflammatory functions and gene expression^13,26^. Blocking glycolysis with 2-deoxy-glucose (2-DG), or HIF-1 function with Acriflavine, an inhibitor that blocks the dimerization between HIF-1α and HIF-1β^27^, inhibited the expression of LPS-induced inflammatory and glycolysis genes in peritoneal macrophages (PMφs) (Supplementary Fig. 1A and 1B). We previously showed that cholesterol loading impaired HIF-1α-mediated glycolysis and the expression of glycolytic genes in LPS-stimulated PMφs^22^. To elucidate the mechanism, we first confirmed that cholesterol loading of PMφs inhibited LPS-induced HIF-1α function. Accumulation of cholesterol in lipid droplets increased intracellular lipid content in PMϕ (Supplementary Fig. 1C and 1D), impaired nuclear accumulation of HIF-1α and HIF-2α, and modestly increased basal HIF-2α levels, but this increase was not statistically significant (Fig. 1A). Similar findings were also observed in RAW 264.7 cells, a Mφ cell line (Supplementary Fig. 1E). Cholesterol loading also reduced HIF-1α mRNA levels after LPS stimulation in PMφs (Supplementary Fig. 1F). In terms of HIF-1α function, cholesterol loading of BMDMφs impaired LPS-induced glycolysis, as determined by a glycolysis stress test (GST) measurements of extracellular acidification rate (ECAR) using a Seahorse analyzer (Fig. 1B). Similar results were obtained from BMDMφs loaded with oxLDL (Supplementary Fig. 1G).

The HIF-1α protein is unstable under normoxic conditions and its half-life is approximately 5 min^17^. Upregulation of protein stability is the primary way cells increase HIF-1α expression and function. To determine how cholesterol loading of PMφs reduced LPS-induced HIF-1α protein levels, we first assessed if cholesterol loading modulates HIF-1α stability. We found that cholesterol loading accelerated HIF-1α degradation in PMφs stimulated with LPS for 8 h then treated with cycloheximide (CHX), a protein synthesis inhibitor (Fig. 1C). In the classical model of HIF-1α degradation, PHDs hydroxylate key proline resides in the ODD domain of HIF-1α, which serve as docking sites for VHL ubiquitin ligase complex that mediates HIF-1α ubiquitination and proteasomal degradation^28–30^. We therefore investigated if cholesterol loading modulates the activity of PHDs. RAW264.7 cells were transfected with a luciferase reporter construct with the ODD domain of HIF-1α fused to the luciferase (ODD-luciferase), so that the stability of luciferase expression is regulated by PHDs^28–31^. Using this reporter construct, we found that LPS stimulation increased the expression of luciferase, consistent with the suppression of the enzymatic activity of PHDs (Fig. 1D, left). Cholesterol loading significantly impaired the induction of luciferase (Fig. 1D, right), which suggests that LPS stimulation of cholesterol loaded cells suppressed the activity of PHDs to a lesser extent. Taken together, our data suggest that the activity of PHDs is higher in LPS-stimulated PMφs with accumulated cholesterol or oxLDL. No changes to the protein levels of PHD1, PHD2 and PHD3 were found in cholesterol loaded PMφs at baseline or after LPS stimulation (Supplementary Fig. 1H).

We next investigated how cholesterol loading of Mφs modulates the activity of PHDs. Among all the co-factors that regulate PHDs, reactive oxygen species (ROS) has emerged as a critical regulator due to its ability to oxidize the catalytic ferrous ion of PHDs (Fe^+2^) into ferric iron^32^. Indeed, past studies including ours have shown that blocking LPS-induced ROS significantly impaired HIF-1α levels^22^. We used CellROX to quantify total ROS levels in PMφs and found that cholesterol loading impaired LPS-induced ROS (Fig. 1E). Similar data were obtained with MitoSOX (Supplementary Fig. 1I), suggesting that cholesterol loading also reduced mitochondrial-derived ROS that is induced by LPS. We then explored how cholesterol loading of PMφs suppresses LPS-induced ROS. Since steady-state ROS levels are dependent on both the rate of production and detoxification, we examined the expression of major players that synthesize ROS in LPS-activated PMφs, including  NOX2 and NOS2, in which their genetic deficiency resulted in impaired LPS-induced ROS levels (Supplementary Fig. 1J). Using qPCR and immunoblotting, we found that LPS-induced mRNA (Fig. 1F) and protein (Fig. 1G) expression of NOX2 and NOS2 were inhibited by cholesterol loading. NOX2 protein expression was modestly increased by cholesterol loading alone, but this increase was not statistically significant (Fig. 1G). Taken together, our results show that cholesterol loading increases the activity of PHDs and enhances HIF-1α degradation in LPS-stimulated Mφs.

### Cholesterol loading of Mφs impairs HIF-1 function by decreasing HIF-1α stability in a *Vhl*-dependent manner and by reducing the transactivation capacity of HIF-1α

**Figure 2:**
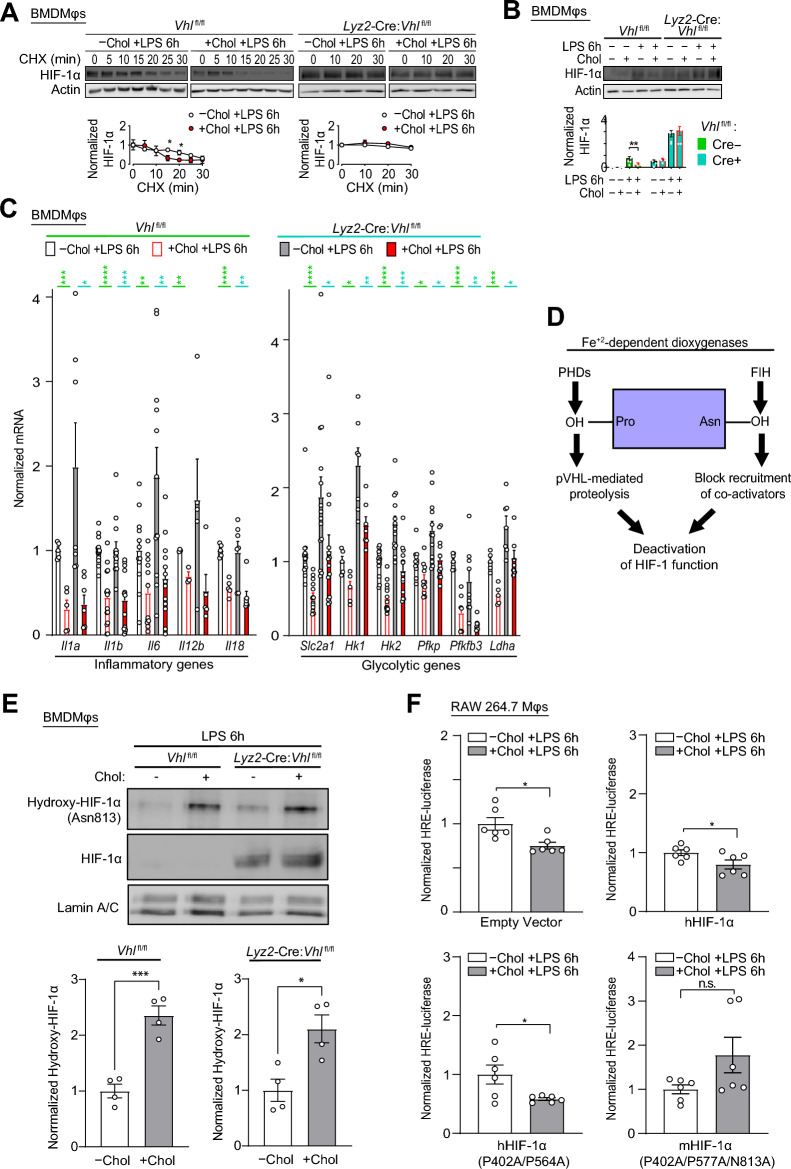
Cholesterol loading of Mφs impairs HIF-1 function by decreasing HIF-1α stability in a *Vhl*-dependent manner and reducing its transactivation capacity. (**A**, **B**) Assessment of HIF-1α protein stability (**A**) and abundance (**B**). Representative immunoblots and quantification showing HIF-1α protein accumulation in BMDMφs derived from *Vhl*^fl/fl^ and *Lyz2-*Cre:*Vhl*^fl/fl^ mice with and without cholesterol loading and LPS stimulation (6 h). (**A**) Lysates were harvested after cycloheximide (CHX) treatment (0–30 min) and HIF-1α values are normalized to the corresponding actin and the pre-CHX time point (assigned a value of 1, n = 3–4). (**B**) CHX was not used. HIF-1α values are normalized to the corresponding actin (n = 6). (**C**) qPCR analysis of inflammatory and glycolysis gene mRNA expression in BMDMφs derived from *Vhl*^fl/fl^ and *Lyz2*-Cre:*Vhl*^fl/fl^ mice. Cells with and without cholesterol loading were stimulated with LPS for 6 h (n = 3–16). (**D**) Schematic illustrating how PHDs and FIH, which are Fe^+2^-dependent dioxygenases, decrease HIF-1 function. (**E**) Representative immunoblots and quantification of nuclear HIF-1α Asn813 hydroxylation and HIF-1α protein abundance. *Vhl*^fl/fl^ and *Lyz2*-Cre:*Vhl*^fl/fl^ BMDMφs with and without cholesterol loading were stimulated with LPS for 6 h. Asn813 hydroxylation data are normalized to the corresponding genotype HIF-1α –Chol group (assigned a value of 1, n = 4). (**F**) Assessment of HIF-1α transcription. RAW 264.7 cell lines expressing various HIF-1α mutant constructs and an HRE-luciferase reporter were cultured with or without cholesterol and stimulated with LPS for 6 h. For each HIF-1α construct, data are normalized to the –Chol group (assigned a value of 1, n = 6). The mean ± SEM is plotted in all graphs. Significant differences are determined by a two-way ANOVA with Bonferroni correction (**A**, **B**) and unpaired Student’s *t*-test (**C**, **E**, **F**). (*P < 0.05, **P < 0.01, ***P < 0.001, ****P < 0.0001).

We utilized BMDMφs derived from *Lyz2-*Cre:*Vhl*^fl/fl^ mice and Cre-negative littermate controls to investigate the relationship between cholesterol loading and HIF-1α degradation. These BMDMφs are genetically deficient in *Vhl* and thus are incapable of HIF-1α proteasomal degradation. Six hours post-LPS stimulation, cholesterol loading enhanced the degradation of HIF-1α in Cre-negative but not in *Vhl*-deficient BMDMφs (Fig. 2A). Cholesterol loading reduced HIF-1α protein abundance, which was increased by LPS stimulation, in Cre-negative but not *Vhl*-deficient BMDMφs (Fig. 2B). These data show that LPS stimulation increases the abundance of HIF-1α in Mφs and that cholesterol loading reduces HIF-1α primarily by increasing proteasomal degradation. Surprisingly, despite full restoration of HIF-1α stability and expression in *Vhl*-deficient BMDMφs, cholesterol loading still inhibited the expression of most LPS-induced inflammatory and glycolysis genes, although to a lesser extent compared to Cre-negative littermates (Fig. 2C and Supplementary Fig. 2A).

We next assessed the transcription function of HIF-1α in cholesterol loaded *Vhl*-deficient BMDMφs. Pyruvate kinase M2 (PKM2) can transition from tetramer to dimer and regulate HIF-1α as a transcriptional co-activator^33^. We investigated the phosphorylation of PKM2 Tyrosine 105 (Y105), which correlates with dimer formation, but found that cholesterol loading did not alter Y105 phosphorylation in PMφs (Supplementary Fig. 2B). Studies in the hypoxia field have highlighted the importance of HIF-1α transactivation capacity, specifically the ability of HIF-1α to recruit co-activators, such as p300/CBP^33,34^. Factor inhibiting HIF (FIH) is a key enzyme that regulates the transactivation capacity of HIF-1α. By hydroxylating an asparagine residue of HIF-1α, FIH blocks the recruitment of co-activators^21^. Given that both FIH and PHDs belong to the same family of Fe^+2^-dependent dioxygenases (Fig. 2D), and that cholesterol loading increased the activity of PHDs (Fig. 1D), this suggests that cholesterol loading also increases the activity of FIH, hence blocking HIF-1α function by inhibiting co-activator recruitment. Indeed, we have previously generated RAW264.7 Mφ cell lines that stably expressed different HIF-1α mutants and found that the inhibition of LPS-induced inflammatory and glycolysis gene expression by cholesterol loading was rescued only in cells transfected with HIF-1α with mutated hydroxylation sites targeted by both PHDs and FIH^22^. However, to validate that the activity of FIH was directly induced by cholesterol loading, we assessed the hydroxylation levels of HIF-1α on Asparagine 813 (Asn813), the position where FIH hydroxylates mouse HIF-1α, using a mouse monoclonal antibody that recognizes this hydroxylation site^35^. We found that cholesterol loading increased the hydroxylation of HIF-1α Asn813 in LPS-stimulated BMDMφs derived from both control (Cre-negative *Vhl*^fl/fl^) and *Vhl*-deficient (*Lyz2-*Cre*:Vhl*^fl/fl^) mice (Fig. 2E). As expected, the abundance of nuclear HIF-1α protein was lower in control relative to *Vhl*-deficient BMDMφs and cholesterol loading reduced nuclear HIF-1α protein in control but not *Vhl*-deficient BMDMφs (Fig. 2E). Similar HIF-1α Asn813 hydroxylation data were obtained from control and *Vhl*-deficient BMDMφs following loading with oxLDL (Supplementary Fig. 2C). We next directly assessed the transcription function HIF-1α by using a luciferase reporter construct in which the hypoxia response element (HRE, the consensus sequence that HIF-1α binds to) is fused to a luciferase reporter (HRE-luciferase); therefore, the expression of luciferase is under the direct regulation of HIF-1α^36^. This HRE-luciferase reporter was transfected into our RAW264.7 Mφ cell lines that stably expressed different HIF-1α mutants: HA-hHIF-1α WT control, HA-hHIF-1α (P402A/P564A) and Myc-mHIF-1α (P402A/P577A/N813A). Cholesterol loading significantly reduced luciferase expression in cell lines stably transfected with empty vector, HA-hHIF-1α WT control and HA-hHIF-1α (P402A/P564A) constructs (Fig. 2F). In contrast, luciferase expression was not altered by cholesterol loading in the cell line stably transfected with Myc-mHIF-1α (P402A/P577A/N813A) triple mutant which blocks both PHD and FIH functions (Fig. 2F). Collectively, our data show that cholesterol loading of Mφs impairs HIF-1α function by increasing the activity of PHDs as well as FIH, thereby decreasing both HIF-1α stability and transactivation capacity, respectively.

### Cholesterol loading of Mφs induces oxidative stress and enhances a NRF2-dependent responses after LPS stimulation

**Figure 3:**
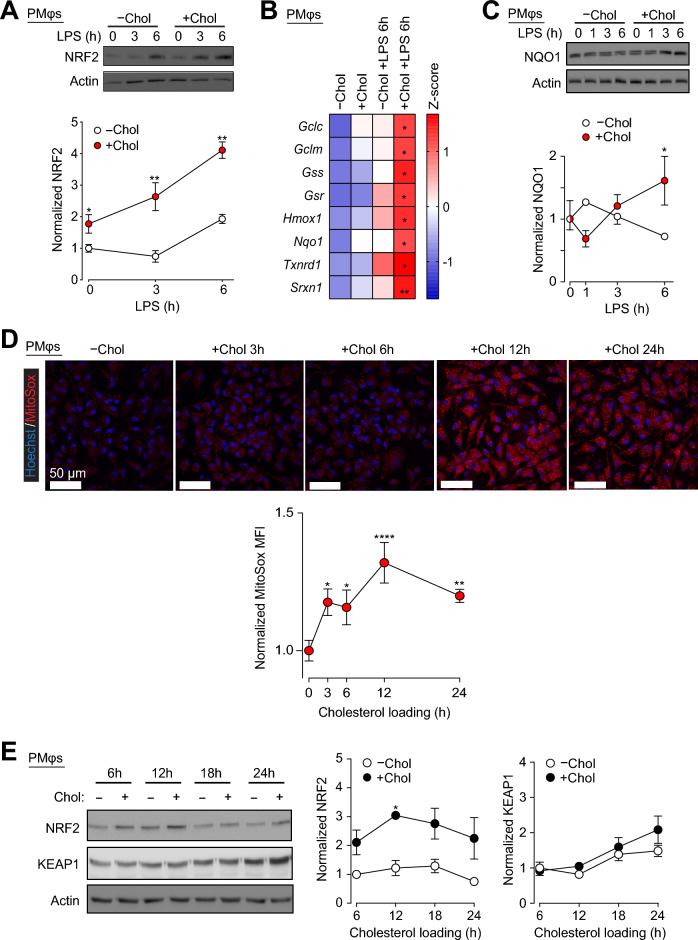
Cholesterol loading-induces oxidative stress in Mφs and after LPS stimulation enhances NRF2-dependent responses. (**A**) Representative immunoblots and quantification of NRF2 protein expression in PMφs with and without cholesterol loading. A 0–6 h time course after LPS stimulation was performed. NRF2 values are normalized to the corresponding actin and the 0 h LPS time point in the –Chol group (assigned a value of 1, n = 3). (**B**) A heatmap showing qPCR analysis of NRF2-regulated gene mRNA expression in PMφs with and without cholesterol loading and LPS stimulation (6 h, n = 3–12). (**C**) Representative immunoblots and quantification of NQO1 protein in PMφs ± cholesterol loading. A 0–6 h time course after LPS stimulation was performed and values are normalized to -Chol and 0 h LPS time point (assigned a value of 1, n = 3). (**D**, **E**) The accumulation of cholesterol increases mitochondrial ROS (**D**) and NRF2 protein abundance (**E**) in PMφs in a time-dependent manner. (**D**) Representative images and quantification show MitoSox staining (red) at different time points after addition of cholesterol to cultured PMφs. Data are normalized to cells cultured for 24 h without cholesterol (–Chol image and 0 h Chol time point in the graph, assigned a value of 1, n = 3–10). Scale bars, 50 µm. (**E**) Representative immunoblots and quantification of NRF2 and KEAP1 protein. Data are normalized to actin and the –Chol group 6 h time point (assigned a value of 1, n = 3). The mean ± SEM is plotted in all graphs. Significant differences are determined by a one-way (**D**) or two-way ANOVA (**A**–**C**, **E**) with Bonferroni correction (*P < 0.05, **P < 0.01, ****P < 0.0001).

How cholesterol loading of Mφs impairs LPS-induced ROS levels remains unclear (see Fig.1E). While we previously showed that cholesterol loading of PMφs inhibited the expression of  NOX2 and NOS2 (Fig. 1F, G), thereby reducing ROS synthesis, but this fails to explain the reduction of mitochondrial ROS (Supplementary Fig. 1I). A possible explanation is that cholesterol loading induces a robust ROS detoxification response, thereby lowering ROS levels across all cellular compartments. We previously showed that oxLDL loading of Mφs enhanced LPS-induced NRF2-dependent antioxidative defense response^22^, but it is unknown if this mechanism is triggered by cholesterol loading. To explore this, we assessed NRF2 protein in PMφs by immunoblotting and found that cholesterol loading increased the abundance of NRF2 before and after LPS stimulation (Fig. 3A). Next, we assessed the expression of NRF2-regulated detoxification genes, including NQO1, and found that in LPS-stimulated PMφs cholesterol loading significantly increased the expression of mRNA (Fig. 3B, multiple genes) and protein (Fig. 3C, NQO1). Taken together, these results support that notion that cholesterol loading of Mφs enhances LPS-induced NRF2-dependent antioxidative response, analogous to oxLDL loading^22^.

The induction of NRF2 protein levels by cholesterol loading prior to LPS stimulation (Fig. 3A) is of particular interest as it suggests that the redox environment is modulated by cholesterol loading prior to LPS stimulation. In fact, we previously showed that oxLDL loading of Mφs alone could induce oxidative stress and NRF2 activation^8,22^. To determine if cholesterol loading induces a similar phenomenon, we assessed mitochondria-derived ROS with MitoSOX and found that cholesterol loading of PMφs induced ROS in a time-dependent manner (Fig. 3D). The induction of mitochondrial ROS correlated with an increase in NRF2 protein without significant changes in KEAP1 protein expression (Fig. 3E). These data suggest that loading of Mφs with cholesterol induces oxidative stress and activates NRF2 even prior to LPS stimulation.

### The NRF2 response induced by cholesterol loading impairs HIF-1 function induced by LPS stimulation

**Figure 4:**
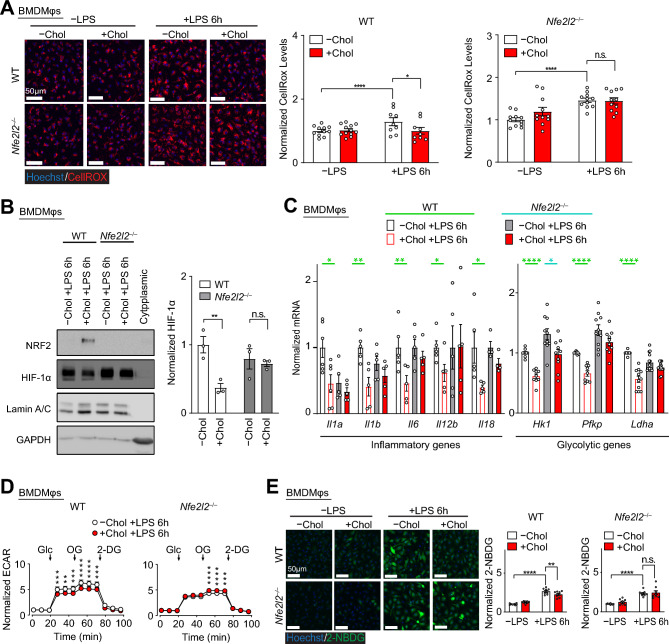
The NRF2 response to cholesterol loading impairs LPS-induced HIF-1α-dependent glycolytic reprogramming. (**A**) Effect of cholesterol accumulation and LPS stimulation (6 h) on ROS in BMDMφs with NRF2 deficiency. Representative images and quantification of total ROS (CellROX, red) in cells derived from *Nfe2l2*^+/+^ (WT) and *Nfe2l2*^–/–^ littermates are shown. Data are normalized to BMDMφs without cholesterol and LPS (assigned a value of 1, n = 9–12). Scale bars, 50 µm. (**B**) Representative immunoblots and quantification of nuclear NRF2 and HIF-1α proteins in WT and *Nfe2l2*^*−/−*^ BMDMφs with and without cholesterol loading and LPS stimulation (6 h). Data are normalized to BMDMφs without cholesterol (assigned a value of 1, n = 3). (**C**) qPCR analysis of inflammatory and glycolysis gene mRNA expression in WT and *Nfe2l2*^*−/−*^ BMDMφs with and without cholesterol loading and with LPS stimulation (6 h). Data are normalized to BMDMφs without cholesterol (assigned a value of 1, n = 3–6). (**D**) Glycolysis stress tests in WT and *Nfe2l2*^*−/−*^ BMDMφs with and without cholesterol and LPS stimulation (6 h). ECAR values normalized to baseline (assigned a value of 1) are plotted (n = 10–12). (**E**) Representative confocal microscopic images and quantification of 2-NBDG in ± cholesterol loaded BMDMφs derived from WT and *Nfe2l2*^*−/−*^ mice post 6 h of LPS stimulation. Data are normalized to BMDMφs without cholesterol and LPS for each genotype (assigned a value of 1, n = 9). The mean ± SEM is plotted in all graphs. Significant differences are determined by a two-way ANOVA with Bonferroni correction (*P < 0.05, **P < 0.01, ***P < 0.001, ****P < 0.0001).

We next determined if the NRF2 antioxidative response induced by cholesterol loading directly impaired HIF-1 function after LPS stimulation. ROS levels after LPS stimulation were assessed in BMDMφs derived from *Nfe2l2*^-/-^ mice (deficient in the gene encoding NRF2). In contrast to WT BMDMφs derived from littermates, cholesterol loading did not suppress LPS-induced ROS in *Nfe2l2*^-/-^ BMDMφs (Fig. 4A), which suggests that cholesterol loading of Mφs primarily suppressed LPS-induced ROS through detoxification. To evaluate if NRF2-mediated detoxification underlies the impairment of HIF-1 function, we then examined HIF-1α abundance in the nucleus and found that cholesterol loading did not significantly reduce nuclear HIF-1α in *Nfe2l2*^*-/-*^ BMDMφs, in contrast to WT littermate controls (Fig. 4B). Furthermore, we also found that *Nfe2l2*^*-/-*^ BMDMφs were resistant to the inhibitory effects of cholesterol loading on the expression of inflammatory and glycolysis genes (Fig. 4C and Supplementary Fig. 3A). Similar findings were also observed on the protein level (Supplementary Fig. 3B). Finally, we assessed the glycolytic function of *Nfe2l2*^*−/−*^ BMDMφs using a glycolysis stress test and found that cholesterol loading did not suppress LPS-induced glycolysis in contrast to WT controls (Fig. 4D). Cholesterol loading also did not reduce glucose uptake, measured using a fluorescently-labelled glucose analog 2-NBDG (2-(N-(7-Nitrobenz-2-oxa-1,3-diazol-4-yl) Amino)-2-Deoxyglucose), in LPS stimulated *Nfe2l2*^*-/-*^ BMDMφs in contrast to WT cells (Fig. 4E). Collectively, our results demonstrated that cholesterol loading of Mφs suppresses HIF-1α-mediated inflammatory and glycolytic responses in a NRF2-dependent manner.

### Cholesterol loading induces NRF2 stabilization and primes Mφs for an enhanced NRF2 response after LPS stimulation

**Figure 5:**
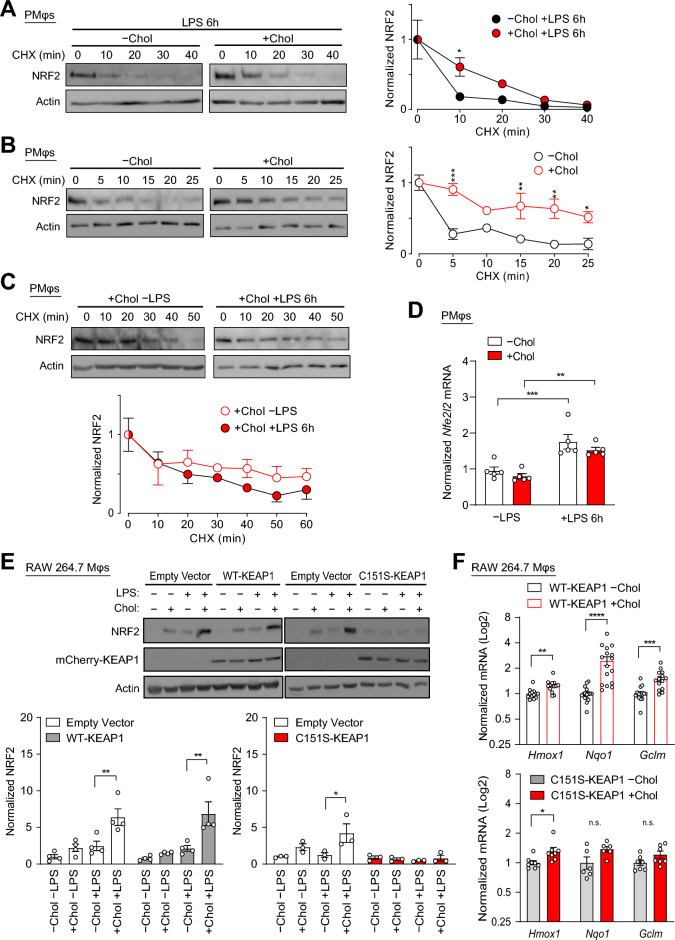
The primary NRF2 response to cholesterol loading primes an enhanced secondary NRF2 response to LPS stimulation. (**A**–**C**) Assessment of NRF2 protein stability in PMφs. Time course experiments were performed after cycloheximide (CHX) treatment to block new protein synthesis. Representative immunoblots and quantification are shown. NRF2 values are normalized to the corresponding actin and the pre-CHX time point (assigned a value of 1). (**A**) Effect of cholesterol loading after LPS stimulation (6 h, n = 3–4). (**B**) Effect of cholesterol loading without LPS stimulation (n = 3). (**C**) Effect of LPS stimulation (6 h) on cholesterol loaded PMφs (n = 3). (**D**) qPCR analysis of *Nfe2l2* mRNA expression in PMφs with and without cholesterol loading and LPS stimulation (6 h). Data are normalized to the –Chol –LPS group (n = 5–8). (**E**, **F**) Effect of a KEAP1 C151S mutation on NRF2 expression and NRF2-regulated mRNA expression in RAW264.7 cells transfected with empty vector, WT-mCherry-KEAP1 and C151S-mCherry-KEAP1 constructs. (**E**) Representative immunoblots and quantification of NRF2 and mCherry-KEAP1 in RAW264.7 cells with and without cholesterol loading and 6 h LPS stimulation. Data are normalized to -Chol and -LPS for each group  (n = 3–4). (**F**) NRF2-regulated gene (*Hmox1*, *Nqo1* and *Gclm*) mRNA expression assessed by qPCR in cells with and without cholesterol loading (n = 13–16). Data are normalized to -Chol for each gene. The mean ± SEM is plotted in all graphs. Significant differences are determined by a two-way ANOVA with Bonferroni correction (**A**–**E**) or unpaired Student’s *t*-test (**F**) (*P < 0.05, **P < 0.01, ***P < 0.001, ****P < 0.0001).

While we showed in Fig. 3 that cholesterol loading activates the induction of a primary NRF2 response, it remains to be determined how this primary response leads to an enhanced secondary response after LPS stimulation. We first investigated the role of p62, a protein that participates in a positive NRF2 feedback loop, i.e., NRF2 regulates the transcription of p62, which mediates the autophagic degradation of KEAP1, an adaptor of Cullin 3-based E3 ubiquitin ligase that negatively regulates NRF2^37^. Cholesterol loading of PMφs did not significantly alter p62 and KEAP1 protein abundance before or after LPS treatment (Supplementary Fig. 4A), suggesting that cholesterol loading does not affect the NRF2-p62 feedback loop. We then investigated if NRF2 stabilization after cholesterol loading of PMφs is due to modulation of NRF2 degradation, as the stability of NRF2 is the primary mechanism behind the regulation of its expression and thus function. In a CHX chase assay, cholesterol loading of PMφs significantly increased the stability of NRF2 6 h after LPS stimulation (Fig. 5A). Loading of PMφs with cholesterol alone was sufficient to increase NRF2 stability (Fig. 5B), this suggested that the enhanced NRF2 response induced by cholesterol loading is independent of LPS stimulation. Indeed, LPS stimulation did not further increase NRF2 stability in cholesterol loaded PMφs (Fig. 5C). We obtained similar data from PMφs loaded with oxLDL (Supplementary Fig. 4B-D). Although LPS did not further stabilize NRF2 in PMφs loaded with cholesterol or oxLDL, LPS stimulation increased NRF2 protein expression and function in cholesterol loaded Mφs (Fig. 3A–C). A possible explanation for this is that LPS stimulation increases NRF2 transcription and mRNA expression. To confirm this, we measured *Nfe2l2* (NRF2) mRNA expression and found LPS significantly increased its expression independent of cholesterol loading (Fig. 5D). Collectively, we demonstrated that cholesterol loading of PMφs stabilized NRF2 and increased NRF2 protein expression prior to LPS stimulation. This baseline difference in protein expression was further accentuated by LPS-induced upregulation of NRF2 protein synthesis due to increased *Nfe2l2* transcription and accounted for the enhanced NRF2 response observed in LPS-stimulated cholesterol-loaded PMφs.

Finally, we investigated the mechanisms underlying NRF2 stabilization by cholesterol-induced oxidative stress. KEAP1, a negative regulator of NRF2 that constitutively mediates its proteasomal degradation, senses cellular oxidative stress through reactive free thiol groups found on multiple cysteine residues^24^. Amongst these, cysteine 151 (C151) has been characterized the most and was found to be critical in the regulation of KEAP1 function, i.e., increased NRF2 expression in response to oxidative insults^38^. To investigate the possibility that cholesterol loading induces NRF2 stabilization by modulating KEAP1 C151, we transfected RAW 264.7 Mφs with WT-mCherry-KEAP1 and mutant cysteine 151 to serine (C151S)-mCherry-KEAP1 cDNA constructs^38^ and assessed NRF2 protein expression after cholesterol loading and LPS stimulation. NRF2 expression was significantly increased by cholesterol loading and LPS stimulation in cells transfected with empty vector and WT-mCherry-KEAP1 construct, but this increase was abrogated in cells transfected with C151S-mCherry-KEAP1 (Fig. 5E). We next used qPCR to assess the effect of cholesterol loading on the mRNA expression of NRF2 target genes, including *Hmox1*, *Nqo1* and *Gclm*. Cholesterol loading induced mRNA expression in cells transfected with WT-mCherry-KEAP1, but this was dampened in cells transfected with C151S-mCherry-KEAP1 (Fig. 5F). Collectively, our data suggest that cholesterol loading impairs the sensory function of KEAP1 cysteine residues, especially C151, and this leads to basal stabilization of NRF2. This basal stabilization together with LPS-induced synthesis of NRF2 leads to an enhanced antioxidative defense response observed in cholesterol-loaded Mφs after LPS stimulation.

## Discussion

Foam cell formation is a typical feature of atherosclerosis, but the link between lipid loading of Mφs and inflammation remains elusive. While some studies have suggested that the formation of cholesterol crystals that activate the NLRP3 inflammasome^4^ and novel signaling by oxLDL promote inflammation^39^, other reports have shown that cholesterol loading impairs Mφ inflammatory responses in an LXR-dependent^5^ and -independent manner^6–8^. Our previous research supported the notion that lipid loading is not an inflammatory process per se as the accumulation of oxLDL in Mφs alone was insufficient to induce inflammation^7,8,22^. Mechanistically, when oxLDL loaded Mφs were stimulated by LPS, HIF-1α-dependent glycolysis and inflammation were suppressed by an upregulated NRF2-dependent antioxidative response^22^. It was unknown if loading of Mφ with free cholesterol triggered a similar mechanism. Furthermore, how lipid loading induces oxidative stress and primes for an enhanced NRF2-dependent antioxidative response was poorly understood. In the current study, we demonstrated that LPS stimulation of Mφs loaded with free cholesterol increases the activity of PHDs and FIH, thereby reducing HIF-1α stability and transactivation capacity respectively, eventually leading to the suppression of HIF-1α-dependent transcription of glycolysis genes. Even prior to LPS stimulation, cholesterol loading of Mφs induced oxidative stress and the modification of KEAP1 cysteine residues, which function as oxidative stress sensors (see below), led to the stabilization of NRF2. The pre-stabilization of NRF2 combined with the increased levels of NRF2 mRNA and protein upon LPS stimulation led to an enhanced NRF2 antioxidative response that ultimately impaired HIF-1α-dependent glycolytic and inflammatory responses. These data suggest that the immunometabolic adaptation of Mφs to loading with free cholesterol or oxLDL is similar, although the routes of lipid uptake are different. Free cholesterol is passively taken up by the plasma membrane, while oxLDL is engulfed and enters the endolysosomal pathway. In both instances, cholesterol is eventually redistributed throughout the cell, and perhaps the metabolism of cholesterol, like the metabolism of fatty acids observed in oxLDL-loaded Mφs^8^, contributes to the production of mitochondrial free radicals (Fig. 3D) without triggering an inflammatory response.

The role of FIH is well-established in the hypoxia research field^21^; however, it is less appreciated in the field of immunometabolism. Mice that were genetically deficient of FIH were previously generated and found that it plays a negative role in regulating oxidative and glycolytic metabolism in murine embryonic fibroblasts^40^. This agrees with the established notion that FIH and PHDs act synergistically to regulate HIF-1-dependent metabolism. Similarly, our past investigation has also shown that cholesterol loading failed to inhibit the expression of LPS-induced inflammatory and glycolysis genes only when cells expressed HIF-1α mutants that blocked the hydroxylation from both FIH and PHDs^22^. However, there was a lack of evidence showing that cholesterol loading directly activates FIH activity. Therefore, in this study, we have utilized an antibody that specifically recognizes the asparagine of HIF-1α that FIH hydroxylates and demonstrated that its levels were induced by loading with cholesterol (Fig. 2E) or oxLDL (Supplementary Fig. 2C), thereby strengthening our previous conclusion. Interestingly, it was also discovered in the hypoxia field that FIH has a higher affinity for O_2_ than PHDs^41^, and that HIF-1α is more sensitive to the regulation by FIH than other HIF isoforms, such as HIF-2α^42^. These results implied that despite FIH and PHDs derive from the same family of Fe^+2^-dependent dioxygenases, they regulate HIF-1α differentially, most likely due to intrinsic structural differences. Although O_2_ availability is not the primary factor behind LPS-induced HIF-1α stabilization as all experiments were done in normoxic conditions, the intrinsic differences between FIH and PHDs may still contribute to the highly dynamic regulation of HIF-1α transcriptional output that is required during the course of inflammation.

KEAP1 is a cysteine-rich protein that is a highly conserved across species^24^. KEAP1 is a dominant-negative regular of NRF2 because it constitutively targets NRF2 for proteasomal degradation^24^. Since the free thiol groups found on the cysteine residues can react readily with many electrophilic compounds, including but not limited to ROS^24^ and lipid peroxidation species^43^, they serve as cellular redox sensors and rapidly respond to changes in the redox environment. Upon reaction with electrophiles, these cysteine residues undergo post-translational modifications, thereby changing the conformation of KEAP1 and allowing NRF2 to escape from proteasomal degradation^24^. In our study, we have demonstrated that cholesterol loading of Mφs alone was sufficient to stabilize NRF2 and that LPS stimulation failed to further enhance NRF2 stability (Fig. 5B and C). These data strongly suggest that cholesterol loading and LPS stimulation stabilize NRF2 with the same mechanism, therefore the effect of cholesterol loading on NRF2 stabilization masked the effect of LPS stimulation. In fact, we postulated that cholesterol loading of Mφs have rendered the cysteine sensors of KEAP1 to be dysfunctional prior to LPS stimulation. To test this possibility, we transfected RAW264.7 Mφs with WT and C151S-KEAP1 constructs and subsequently assessed the effects of cholesterol loading on the function of NRF2. As shown in Fig. 5E, immunoblotting analysis showed that cholesterol loading failed to induce NRF2 levels in cells transfected with C151S-KEAP1 constructs. While qPCR analysis still revealed a modest induction of NRF2-targeted genes in cells transfected with C151S-KEAP1 constructs, the extent of induction was less than in cells transfected with WT-KEAP1 constructs (Fig. 5F). These data suggest the possibility that in addition to C151, cholesterol loading may modify other KEAP1cysteine residues. Indeed, C273 and C288 of KEAP1 are also other well-recognized cysteine sensors that are shown to be required for NRF2 ubiquitination and degradation^44^.

In recent years, there is also a growing appreciation to the types of post-translational modifications catalyzed on the cysteine residues on KEAP1, including but not limited to oxidation^45^, S-nitrosation^46^, alkylation^47^, succination^48^ and carbonylation^49^. Amongst these, KEAP1 alkylation is of particular interest as it was recently shown that itaconate-induced alkylation of KEAP1 could induce NRF2-dependent antioxidative response, thereby suppressing Mφ inflammatory responses post LPS stimulation^47^. This led to the development of 4-octyl itaconate, a cell-permeable itaconate derivative, that limits inflammation in many in vivo models^47^. We did not investigate the posttranslational KEAP1 modifications that occur upon loading of Mφs with cholesterol. Nevertheless, we speculate that alkylation is not a likely modification because a past study has shown that KEAP1 alkylation enhances its degradation^50^ but we found that KEAP1 protein expression remained stable in cholesterol loaded Mφs both before (Fig. 3E) and after (Supplementary Fig. 4A) LPS stimulation. Cholesterol loading induced mitochondria-derived oxidative stress (Fig. 3D), which suggests that cholesterol loading may oxidize KEAP1 cysteine residues. The prolonged duration of oxidative stress caused by cholesterol loading (> 12 h) may also allow ROS to react with lipids in cell membranes and thus give rise to lipid peroxidation species, which can then catalyze carbonylation of KEAP1 cysteine residues. Future studies employing mass spectroscopy and structural analysis are warranted to identify the post-translational modifications of KEAP1 cysteines induced by cholesterol loading of Mφs.

## Materials and methods

### Mouse strains

8–12 weeks old mice were used. C57BL/6J (Strain #000,664), B6.129S4(C)-Vhltm1Jae/J (*Vhl*^fl/fl^) (Strain #012933), B6.129X1-Nfe2l2tm1Ywk/J (*Nfe2l2*^*−/−*^) (Strain #017009), B6.129P2-Lyz2tm1(cre)Ifo/J (Strain #004781), B6.129P2-Nos2tm1Lau/J (*Nos2*^*−/−*^) (Strain # 002609) and B6.129S-Cybbtm1Din/J (*Cybb*^*−/−*^) (Strain #002365) mice were purchased from The Jackson Laboratory. *Nfe2l2*^*−/−*^ mice were generated by first crossing with WT C57BL/6J mice, followed by heterozygote intercrossing. *Lyz2-*Cre:*Vhl*^fl/fl^ mice were generated by backcrossing a single *Lyz2-*Cre transgene into *Vhl*^fl/fl^ mice. Breeding for experiments consisted of crosses between Cre-positive and Cre-negative *Vhl*^fl/fl^ mice. All mice were maintained in a pathogen-free, temperature-regulated environment with a 12-h light and dark cycle. All mice used in comparative studies were age-matched, with littermates used as controls. In terms of mouse sexes, both male and female mice were used. Apart from C57BL/6J, all mice were sex-matched across genotypes (Cre-positive and Cre-negative littermates). All mice were fed a normal chow diet (NCD, 16 kcal% fat). All studies were performed under the approval of Animal User Protocols by the Animal Care Committee at the University Health Network according to the guidelines of the Canadian Council on Animal Care. The results in this study were reported in accordance with ARRIVE guidelines.

### Thioglycolate-elicited peritoneal Mφ (PMφ) isolation

Mice were injected intraperitoneally with 1 mL of 4% aged thioglycolate (ThermoFisher Cat#211716) and PMφs were harvested after 4 days by lavage with ice-cold PBS containing 2% FBS. Cells were counted and cultured (37 °C, 5% CO_2_) in DMEM supplemented with 10% FBS, 2 mM l-glutamine, 10,000 U/mL penicillin/streptomycin). Adherent PMφs were used in experiments after 18 h.

### Bone marrow-derived Mφ (BMDMφ) generation

Mice were euthanized in a CO_2_ chamber and bone marrow cells were isolated from leg bones. Cells were cultured (37 °C, 5% CO_2_) in RPMI supplemented with 10% FBS, 2 mM l-glutamine, 10,000 U/mL penicillin/streptomycin, and 40 ng/mL of M-CSF (PeproTech; Cat#AF-315-02) for 7 days. Cells were counted and replated for experiments.

### Transient transfection of RAW264.7 Mφs

RAW264.7 Mφs (2 × 10^6^, ATCC, Cat# ATCC TIB-71) were electroporated (Amaxa® Cell Line Nucleofector® Kit V, LONZA; Cat#VCA-1003) with 2 µg of control plasmid (pcDNA3), WT-mCherry-KEAP1 and C151S-mCherry-KEAP1 plasmids. Transfected cells were seeded in 6-well plates and cultured in recovery medium (DMEM, 20% FBS) for 3 h, then DMEM, 10% FBS for 48 h. The WT-mCherry-KEAP1 and C151S-mCherry-KEAP1 plasmids were a kind gift from the laboratory of Dr. Albena Dinkova-Kostova (University of Dundee).

### Lipid loading, LPS stimulation and inhibitor studies

PMφs, BMDMφs or RAW264.7 Mφs were cultured for 24 h with human medium oxidized low-density lipoprotein (100 μg/mL, Kalen Biomedical Cat#770202) or cholesterol (50 μg/mL, Sigma Cat#C3045), followed by ultrapure LPS stimulation (10 ng/mL, InvivoGen, Cat#tlrl-3pelps) for up to 8 h. Ethanol (0.5%) was used as a carrier control for cholesterol (-Chol). For inhibitor experiments, 2-DG (25 mM) (Sigma, D8375), Acriflavine (2.5 µM) (Sigma, Cat#01673) and TEPP-46 (100-900 µM) (Tocris, Cat#7809) were added 1 h prior to LPS stimulation.

### Immunoblotting

PMφs, BMDMφs or RAW264.7 cells (2 × 10^6^) cultured in 12-well plates, were incubated with or without oxLDL or cholesterol and stimulated with LPS for indicated times. Cells were lysed in ice-cold RIPA buffer (1% NP40, 0.1% SDS, 0.5% deoxycholate in PBS, supplemented with 1 mM PMSF, 1× cOmplete™, EDTA-free Mini Protease Inhibitor Cocktail (Sigma Cat#11873580001) and 1X PhosSTOP™ (Sigma Cat#4906845001)) for 15 min. Protein concentrations in lysates were determined by Protein Assay Dye Reagent (BioRad #5000006), diluted in 2 × Laemmli sample buffer (BioRad Cat#161-0737) with fresh β-mercaptoethanol (BioRad #1610710), and heated at 95 °C for 5 min. Samples (20 μg of protein per lane) were resolved on 8–15% SDS-PAGE gels and transferred to polyvinylidene difluoride membranes (Sigma #IPVH00010) using a wet transfer system. Membranes were blocked with 5% skim milk non-fat powder or 3% BSA (Bioshop #ALB003) in Tris-buffered saline-Tween (TBST) for 1 h at room temperature. Membranes were incubated with primary antibodies overnight: anti-HIF-1α (Cell Signaling Technology (CST)#36169), anti-HIF-2α (Novus Biologicals (NB), NB100-122), anti-Nox2 (Abcam, ab129068), anti-Actin (Sigma, A2066), anti-Lamin A/C (CST#2032), anti-Gapdh (CST#5174), anti-NRF2 (CST#12721), anti-iNOS (Transduction Laboratories, #N32030), anti-hydroxy-HIF-1α (CST#3434), anti-KEAP1 (ThermoFisher, #PA5-99434), anti-p-PKM2 (Y105) (CST#3827S), PKM2 (CST#4053), anti-mCherry (CST#43590), anti-p62 (CST#5114), anti-Nqo1 (CST#62262), anti-IL-1β (CST# #12242), anti-PHD1 (Bethyl Laboratories, Cat#A300-326A), anti-PHD2 (Novus Biologicals, Cat#NB100-138) and anti-PHD3 (Novus Biologicals, Cat#NB100-303), followed by washing and incubation with HRP-conjugated anti-rabbit IgG (CST#7074) or anti-mouse IgG (CST#7076) (22 °C, 1 h). Blots were developed using Immobilon Forte Western HRP substrate (Sigma, WBLUF0100), imaged with Microchemi 4.2 (BioRad) and analyzed with ImageJ. The antibody against hydroxylated Asn813 of HIF-1α was a kind gift from the laboratory of Dr. Myung Kyu Lee (Korea Research Institute of Bioscience and Biotechnology). Phospho-blots were stripped with Western blot stripping buffer (21059X4, ThermoFisher) for 1 h at room temperature. Blots were then washed 3 times (5 min per wash) with 1× TBST buffer and blocked with 5% skim milk (in 1× TBST) for 30 min at room temperature. The stripped blots were then incubated overnight with antibody recognizing the corresponding total protein.

### Cytoplasmic and nuclear subcellular fractionation

PMφs and BMDMφs (6 × 10^6^) were first seeded in 6-well plates, loaded with oxLDL or cholesterol, followed by LPS stimulation as described above. Cells were lysed and scraped with extraction buffer (10 mM HEPES, 1.5 mM MgCl_2_, 10 mM KCl, 1 mM NaF, 0.1% NP40, 1 × cOmplete™, EDTA-free mini protease inhibitor cocktail). Lysates were centrifuged (1200 × g, 4 °C, 10 min) to pellet nuclei and separate cytoplasmic fractions. To purify the nuclear fraction, the pellet was resuspended with extraction buffer and layered on top of a 30% sucrose solution, then centrifuged (3000 × g, 4 °C, 20 min). The supernatant was discarded and nuclei in the pellet were washed once with extraction buffer, and then lysed with a detergent-rich buffer (10 mM HEPES, 1.5 mM MgCl_2_, 10 mM KCl, 1 mM NaF, 0.1% NP40, 1% deoxycholate, 0.1% SDS, 1 × cOmplete™, EDTA-free mini protease inhibitor cocktail).

### Cycloheximide chase assays

PMφs (2 × 10^6^) or BMDMφs were (2 × 10^6^) were cultured in 12-well plates, loaded with cholesterol and stimulated for 6 h or 8 h with LPS (as above). Cycloheximide (CHX, 3 µg/mL, Sigma, Cat#239765) was added to wells in a time-dependent manner.

### Extracellular acidification rate (ECAR) measurement

PMφs (2 × 10^5^) and BMDMφs (3 × 10^5^) were cultured in XF24 well plates (Agilent Technologies, Cat#102342-100), then loaded with cholesterol for 24 h. For glycolysis stress tests, cells were stimulated for 6 h with LPS, washed three times with Seahorse XF DMEM medium (Agilent Technologies, 103334-100) supplemented with 1 mM glutamine and 2 mM pyruvate, and incubated (37 °C, 0% CO_2_) for 30 min prior to the test. During the test, glucose (Agilent Technologies Cat#103577-100), oligomycin A (Cayman Chemical Cat#11342) and 2-deoxyglucose (Sigma Cat#D8375) were added by the XFe24 Seahorse Analyzer (final concentrations were 25 mM, 2 μM and 25 mM, respectively). For real-time ECAR measurements, cells were washed three times with Seahorse XF DMEM medium supplemented with 25 mM glucose, 1 mM glutamine and 2 mM pyruvate, and incubated (37 °C, 0% CO_2_) for 30 min prior to the addition of LPS (10 ng/mL final concentration) or PBS (equal volume) by the XFe24 Seahorse Analyzer.

### Glucose uptake, reactive oxygen species and cholesterol measurement

PMφs (3 × 10^6^) and BMDMs (3 × 10^5^) were first seeded in 35 mm petri dish, with 14 mm microwell (MatTek, P35G-1.5-14-C) or 8-well chamber slides (ThermoFisher Cat#154453) respectively, then cultured with cholesterol overnight. Cells were stimulated with LPS for 6 h the next day, washed three times with pre-warmed HBSS (Wisent, Cat#311-513-CL). For glucose uptake assay, cells were cultured for 1 h at 37 °C, 5% CO_2_ in DMEM without glucose (ThermoFisher #11966025) supplemented with 10% FBS, 2-NBDG (Cayman Chemicals #11046) (100 μg/mL) and 32.4 μM of Hoechst nuclear staining reagent (ThermoFisher #H3570). For reactive oxygen species assay, cells were cultured for 1 h at 37 °C, 5% CO_2_ in HBSS, supplemented with CellRox Orange or Green (10 μM ThermoFisher #C10443 or #C10444) or MitoSox (5 μM, ThermoFisher #M36008) and Hoechst. For BODIPY and MitoTracker staining, cells were cultured for 1 h at 37 °C, 5% CO_2_ in HBSS, supplemented with BODIPY 493/503 (1 μg/mL, ThermoFisher #D3922) and MitoTracker (500 nM, ThermoFisher #M22425). Cells were then washed three times with pre-warmed HBSS. Cells that were stained with CellRox Orange or Green, BODIPY and MitoTracker were imaged live, while cells that were stained with MitoSox Red were fixed with 4% PFA for 1 h at 4 °C prior to imaging. Cells were imaged with Olympus FluoView 1000 Laser Scanning Confocal Microscope (Olympus America) or A1R Confocal microscope with resonant scanner (Nikon). Mean fluorescence intensity measurements represented the ratio of total fluorescence intensity for each field to the number of nuclei in that field. For cholesterol staining with Filipin III, cells cultured on poly-l-lysine-coated glass coverslips were washed three times with PBS and fixed with 3% paraformaldehyde for 1 h at room temperature. After washing three times with PBS on a shaker, cells were incubated with 1 mL of 1.5 mg glycine/mL PBS for 10 min at room temperature, to quench excess fixative. Cells were stained with 0.05 mg/mL Filipin III (Sigma-Aldrich, Oakville, ON) in PBS/10% FBS for 2 h at room temperature. After washing three times with PBS, nuclei were stained for 30 min with NucSpot Live 650 (2× in DMSO, Biotium, Fremont, CA) and plasma membrane, with 20 μg/mL Alexa Fluor-Concanavalin A (Sigma-Aldrich, Oakville, ON). Coverslips were mounted with Dako fluorescent mounting media (Dako, Santa Clara, CA). Images were acquired on a Nikon A1R resonance scanning confocal microscope with a 60× oil immersion objective.

### RNA isolation and real-time (RT) PCR

Total RNA was isolated with E.Z.N.A.® Total RNA Kit I (Omega Cat#R6834-01) and reverse transcription (RT) reactions were performed with High-Capacity cDNA Reverse Transcription Kit (ThermoFisher Cat#4368814) according to manufacturer’s protocol. RT quantitative-PCR (qPCR) was then performed using Roche LightCycler 480 with Luna® Universal qPCR Master Mix (New England Biolabs, Cat#M3003E). Quantification of mRNA was performed by using primers that span over two adjacent exons, quantified using the comparative standard curve method and normalized to hypoxanthine phosphoribosyltransferase (HPRT) as the housekeeping gene. Primer sequences used for qPCR are listed in Table 1:

**Table 1:** PCR primer sequences.

Gene	Forward primer	Reverse primer
*Il1a*	ACGGCTGAGTTTCAGTGAGACC	CACTCTGGTAGGTGTAAGGTGC
*Il1b*	AGTTGACGGACCCCAAAAGA	TGCTGCTGCGAGATTTGAAG
*Il6*	CTCCCAACAGACCTGTCTATACCA	TGCCATTGCACAACTCTTTTCT
*Il12b*	AAGTGGGCATGTGTTCC	TCTTCCTTAATGTCTTCCACTT
*Il15*	GTAGGTCTCCCTAAAACAGAGGC	TCCAGGAGAAAGCAGTTCATTGC
*Il18*	ACAGGCCTGACATCTTCTGC	CCTTGAAGTTGACGCAAGAGT
*Ccl3*	CCCAGCCAGGTGTCATTT	AGTTCCAGGTCAGTGATGTATTC
*Ccl5*	CCTGCTGCTTTGCCTACCTCTC	ACACACTTGGCGGTTCCTTCGA
*Ccl9*	TCCAGAGCAGTCTGAAGGCACA	CCGTGAGTTATAGGACAGGCAG
*Ccl22*	GTGGAAGACAGTATCTGCTGCC	AGGCTTGCGGCAGGATTTTGAG
*Tnfa*	GTAGCCCACGTCGTAGCAAAC	GCACCACTAGTTGGTTGTCTTTGA
*Slc2a1*	GCTTCTCCAACTGGACCTCAAAC	ACGAGGAGCACCGTGAAGATGA
*Hk1*	GAAAGGAGACCAACAGCAGAGC	TTCGTTCCTCCGAGATCCAAGG
*Hk2*	CCCTGTGAAGATGTTGCCCACT	CCTTCGCTTGCCATTACGCACG
*Pfkfb3*	TCATCGAGTCGGTCTGTGACGA	CATGGCTTCTGCTGAGTTGCAG
*Pfkp*	AAGAGGAAACCAAGCAGTGCGC	TTCCTCGGAGTTTCACGGCTTC
*Ldha*	ACGCAGACAAGGAGCAGTGGAA	ATGCTCTCAGCCAAGTCTGCCA
*Gclc*	ACACCTGGATGATGCCAACGAG	CCTCCATTGGTCGGAACTCTAC
*Gclm*	TCCTGCTGTGTGATGCCACCAG	GCTTCCTGGAAACTTGCCTCAG
*Gss*	CCAGGAAGTTGCTGTGGTGTAC	GCTGTATGGCAATGTCTGGACAC
*Gsr*	GTTTACCGCTCCACACATCCTG	GCTGAAAGAAGCCATCACTGGTG
*Hmox1*	CACTCTGGAGATGACACCTGAG	GTGTTCCTCTGTCAGCATCACC
*Nqo1*	GCCGAACACAAGAAGCTGGAAG	GGCAAATCCTGCTACGAGCACT
*Txnrd1*	AGTCACATCGGCTCGCTGAACT	GATGAGGAACCGCTCTGCTGAA
*Srxn1*	TACCAATCGCCGTGCTCATCCG	CCTTTGATCCAGAGGACGTCGA
*Nos2*	TGGCGATCTCAGCAAAAGGTGG	GTACTGTCCCACCTCCATCTTG
*Nox2*	TGGCGATCTCAGCAAAAGGTGG	GTACTGTCCCACCTCCATCTTG
*Nfe2l2*	CAGCATAGAGCAGGACATGGAG	GAACAGCGGTAGTATCAGCCAG
*Hif1a*	CCTGCACTGAATCAAGAGGTTGC	CCATCAGAAGGACTTGCTGGCT
*Hprt*	CAAGCTTGCTGGTGAAAAGGA	TGAAGTACTCATTATAGTCAAGGGCATATC

### Luciferase reporter assay

Using Amaxa® Cell Line Nucleofector® Kit V, RAW264.7 Mφs (2 × 10^6^) were co-transfected with 1 µg of HIF-1α-ODD-luciferase plasmid (Addgene #18965) or 1 µg of HRE-luciferase plasmid (Addgene #26731) and 0.1 µg of CMV–*Renilla* luciferase plasmid. Transfected cells were recovered in DMEM with 20% FBS for 3 h, and media was replaced with DMEM with 10% FBS overnight. Cells were cultured with cholesterol the next day for 24 h and stimulated with LPS the subsequent day for 6 h. Cells were lysed and processed with Promega Dual-Luciferase™ Reporter (DLR™) Assay Systems (Promega Cat#E1910), and both the Firefly and *Renilla* luciferase were quantified by using a GloMax 20/20 luminometer (Promega). CMV-*Renilla* luciferase was used as internal normalization for transfection efficiency.

### Statistical analysis

All the statistical analyses details of experiments can be found in the figure legends. In brief, all figures show pooled data from independent experiments. All experiments were repeated at least three times. The number of biological replicates is listed as the n value. Statistical analyses were performed using the Prism software, unless otherwise specified in the figure legends.

### Supplementary Information

Supplementary Information.

## Data Availability

All data generated or analyzed during this study are included in this published article (and its Supplementary Information files).
