# Supplementary material for: Cholesterol accumulation impairs HIF-1α-dependent immunometabolic reprogramming of LPS-stimulated macrophages by upregulating the NRF2 pathway
Source: Sci Rep. 2024 May 15;14:11162. doi: 10.1038/s41598-024-61493-6 (PMC11096387; doi:10.1038/s41598-024-61493-6)

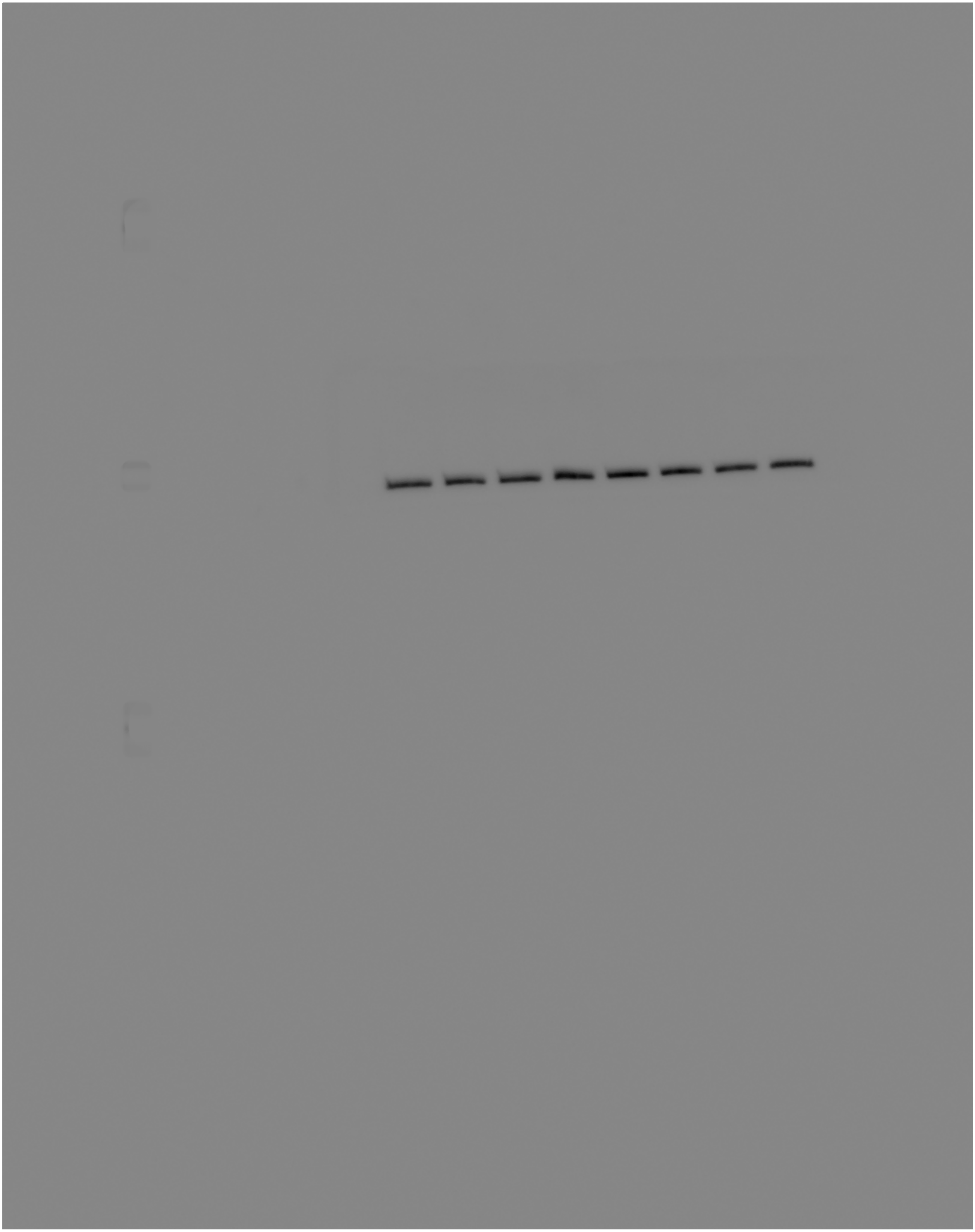

Supplement: Supplementary file 1 — Supplementary Information. [file 41598_2024_61493_MOESM1_ESM.zip › Uncropped blot-Fig3C-actin.pdf]
