# Supplementary material for: Cholesterol accumulation impairs HIF-1α-dependent immunometabolic reprogramming of LPS-stimulated macrophages by upregulating the NRF2 pathway
Source: Sci Rep. 2024 May 15;14:11162. doi: 10.1038/s41598-024-61493-6 (PMC11096387; doi:10.1038/s41598-024-61493-6)
Supplement: Supplementary file 1 — Supplementary Information. [file 41598_2024_61493_MOESM1_ESM.zip › Supplemental_Figures.pdf]

Supplementary Figure 1

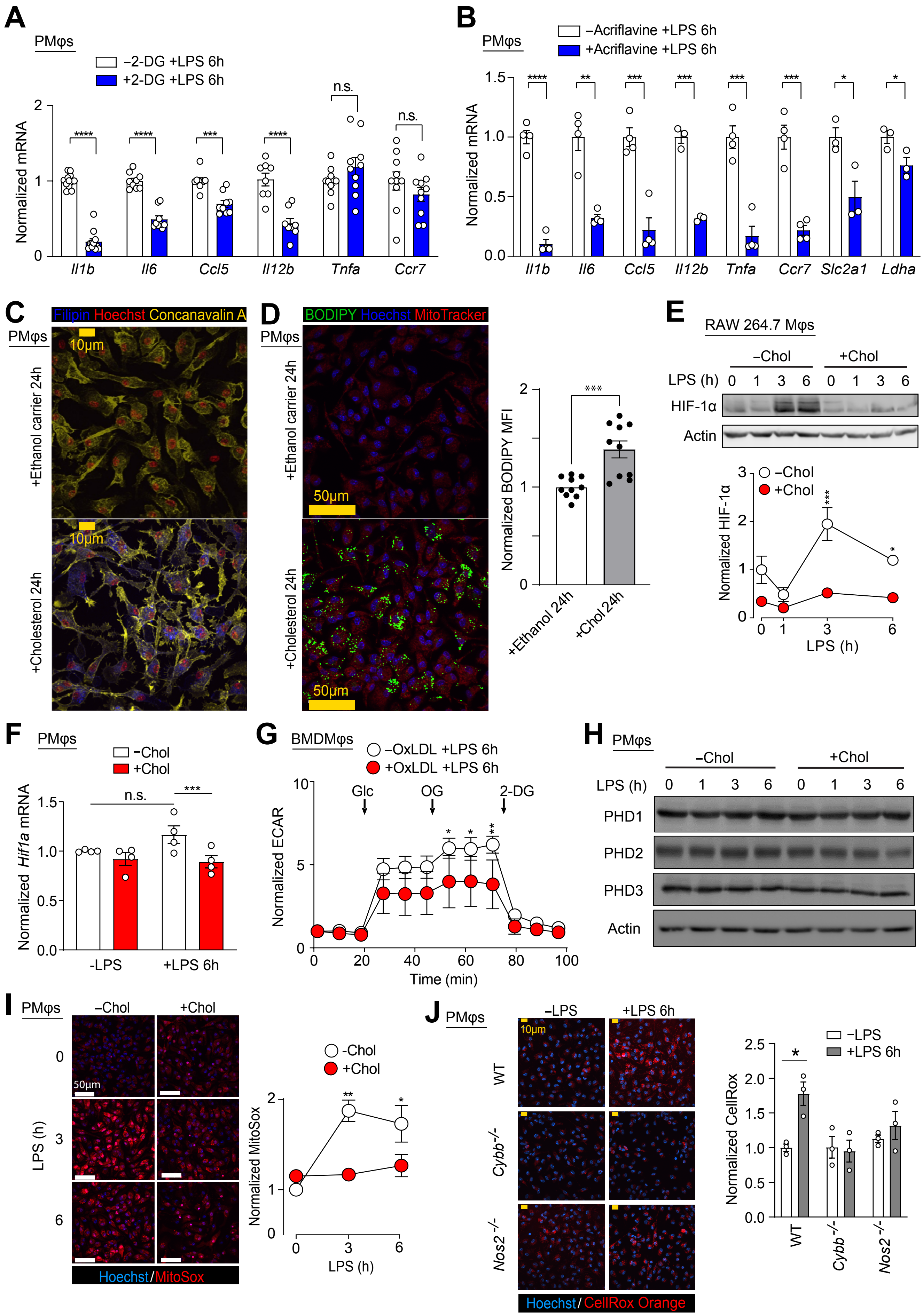

Supplementary Figure 1

(A and B) qPCR analysis of mRNA expression in LPS-stimulated (6 h) PMφs with (+) or without (–) treatment with (A) 2-deoxyglucose (2-DG, n = 8-12) or (B) Acriflavine (n = 3-4). Data are normalized to the corresponding group without treatment (assigned a value of 1). **(C)** Representative confocal microscope images of cultured PMφs treated for 24 h with cholesterol or ethanol carrier. Images show staining of cholesterol with Filipin III (blue), nuclear DNA with Hoechst (red) and cell plasma membranes with Concanavalin A (yellow). Scale bars, 10 μm. **(D)** Representative confocal microscope images of cultured PMφs treated for 24 h with cholesterol or ethanol carrier. Images show staining of cholesterol with BODIPY (green), nuclear DNA with Hoechst (blue) and mitochondria with MitoTracker (red). Scale bars, 50 μm. Shown on the right, is the quantification of BODIPY mean fluorescent intensity (MFI) normalized to the ethanol carrier group (assigned a value of 1, n = 10). **(E)** Representative immunoblots and quantification of HIF-1α. RAW264.7 cells with or without cholesterol (Chol) within 6h LPS stimulation (n = 3). **(F)** qPCR analysis of *Hif1a* mRNA expression in PMφs with or without cholesterol (Chol) loading and 0 or 6 h of LPS stimulation (n = 4). **(G)** Glycolysis stress test showing ECAR (normalized to baseline, assigned a value of 1) in BMDMφs with and without oxLDL loading and 6 h after LPS stimulation (n = 4). Arrows indicate injections of glucose (Glc), oligomycin (OG), and 2-deoxyglucose (2-DG). **(H)** Representative immunoblots of PHD1, PHD2 and PHD3 in PMφs treated with or without cholesterol (Chol) and 0 to 6 h of LPS stimulation (n = 3). **(I)** Effect of cholesterol loading and LPS stimulation (0, 3 and 6 h) on mitochondrial ROS generation in PMφs. Representative confocal microscope images show staining with MitoSOX (red) and Hoechst (blue, nuclei). Quantification of MitoSOX staining is normalized to the group without cholesterol and LPS (assigned a value of 1, n = 12). **(J)** Effect of NOX2 (*Cybb*$^{-/-}$) and Nos2 deficiency on LPS-induced ROS generation in PMφs. Representative confocal microscope images show staining with CellRox Orange (red). Quantification of CellRox staining is normalized to WT cells without LPS stimulation (assigned a value of 1, n = 3). The mean ± SEM is plotted in all graphs. Significant differences are determined by an unpaired Student's *t*-test **(A, B, D, J)** or a two-way ANOVA with Bonferroni correction **(E, F, G, I)** (* $P < 0.05$, ** $P < 0.01$, *** $P < 0.001$, **** $P < 0.0001$).

Supplementary Figure 2

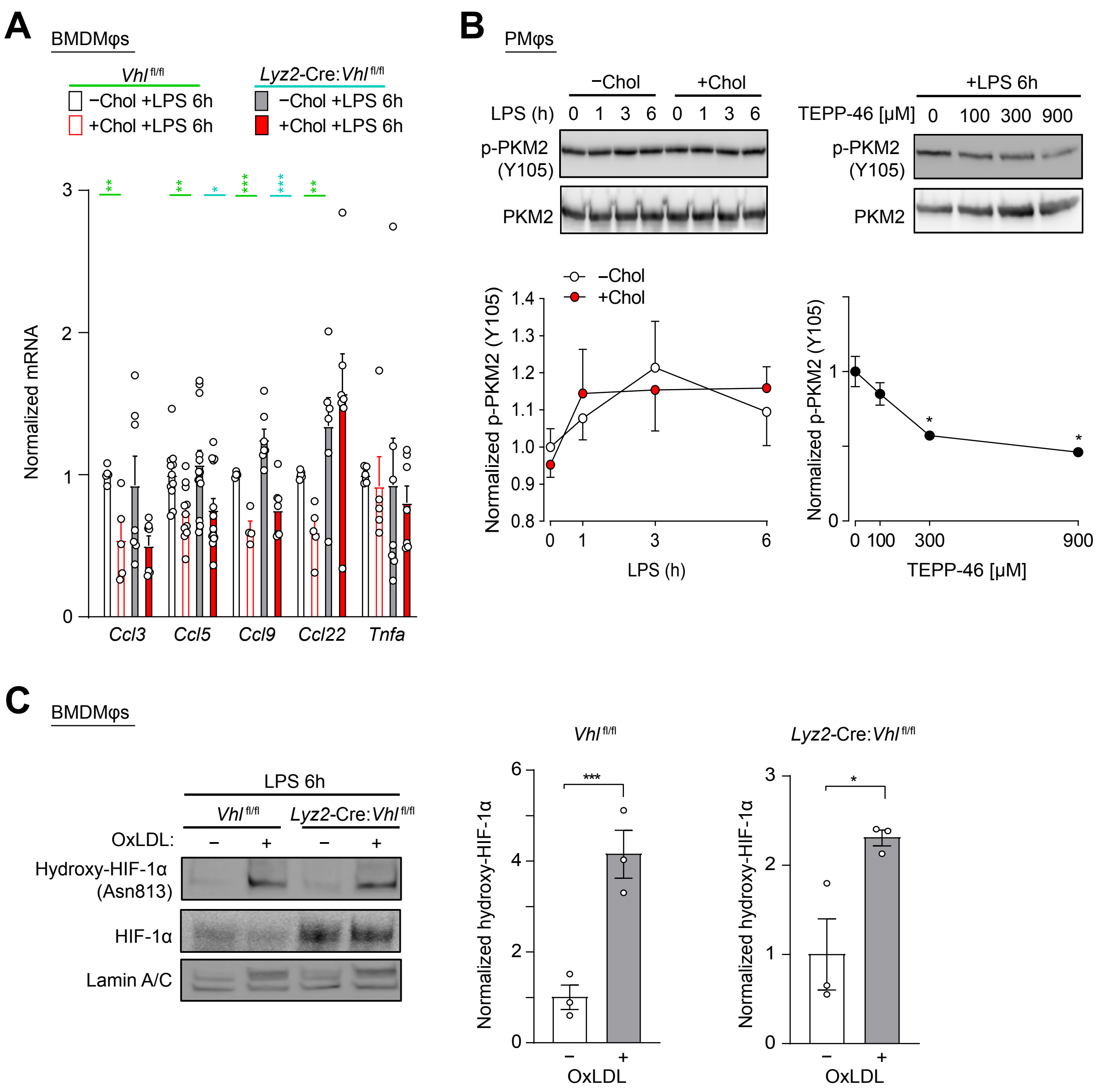

Supplementary Figure 2

(A) qPCR analysis of pro-inflammatory genes in BMDMφs derived from *Vhl*$^{fl/fl}$ and *Lyz2-Cre:Vhl*$^{fl/fl}$ mice with (+) or without (–) cholesterol (Chol) loading and 6 h of LPS stimulation (n = 5-11). **(B)** Representative immunoblots and quantification of PKM2 Tyrosine 105 (Y105) phosphorylation (p) and PKM2 in PMφs with and without cholesterol loading (left). A LPS stimulation time course (0 to 6 h) is shown. Data are normalized to the corresponding PKM2 and 0 h LPS time point of the –Chol group (assigned a value of 1, n = 2). Representative immunoblots and quantification of PKM2 Y105 phosphorylation and PKM2 in PMφs treated with TEPP-46 for 1 h, followed by 6 h of LPS stimulation (right). Data are normalized to the corresponding PKM2 and 0 μM of TEPP-46 (assigned a value of 1, n = 2). **(C)** Representative immunoblots and quantification of HIF-1α Asn813 hydroxylation in the nuclei of LPS-stimulated (6 h) BMDMφs derived from *Vhl*$^{fl/fl}$ and *Lyz2-Cre:Vhl*$^{fl/fl}$ mice with and without oxLDL loading. For each genotype, the data are normalized to the –oxLDL group (assigned a value of 1, n = 3). The mean ± SEM is plotted in all graphs. Significant differences are determined by an unpaired Student's *t*-test **(A, C)** or a one-way ANOVA with Bonferroni correction **(B)** (* $P < 0.05$, ** $P < 0.01$, *** $P < 0.001$).

Supplementary Figure 3

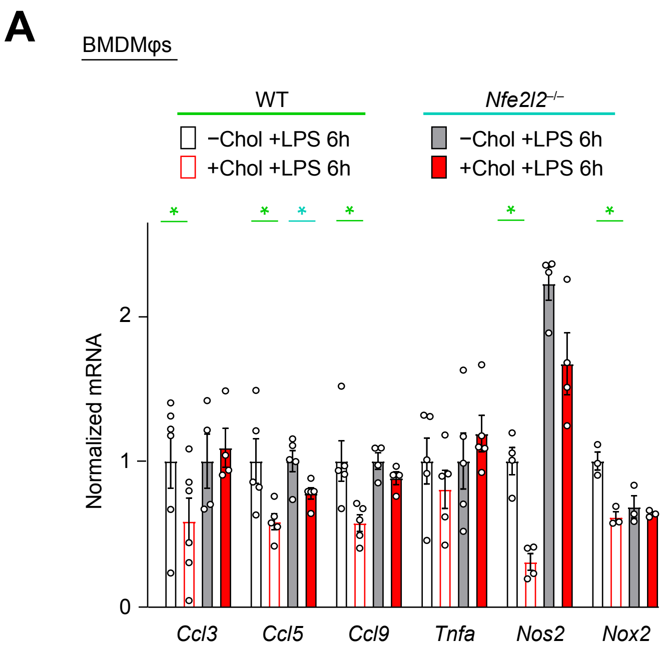

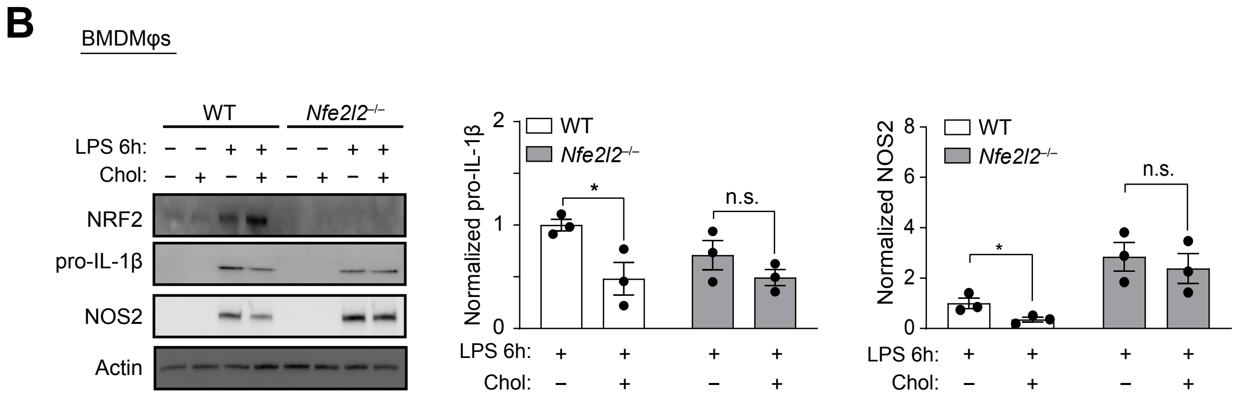

Supplementary Figure 3

(A) qPCR analysis of pro-inflammatory gene mRNA expression in LPS-stimulated (6 h) WT and *Nfe2l2*–/– BMDMφs with (+) and without (–) cholesterol loading (n = 3-6). **(B)** Representative immunoblots and quantification of pro-IL-1β and NOS2 in WT and *Nfe2l2*–/– BMDMφs with and without cholesterol loading and ± LPS stimulation (6 h). Data are normalized to the corresponding actin and the 6 h LPS time point of the WT –Chol group (assigned a value of 1, n = 3). The mean ± SEM is plotted in all graphs. Significant differences are determined by an unpaired Student's *t*-test **(A & B)** (* $P < 0.05$; n.s., not significant).

Supplementary Figure 4

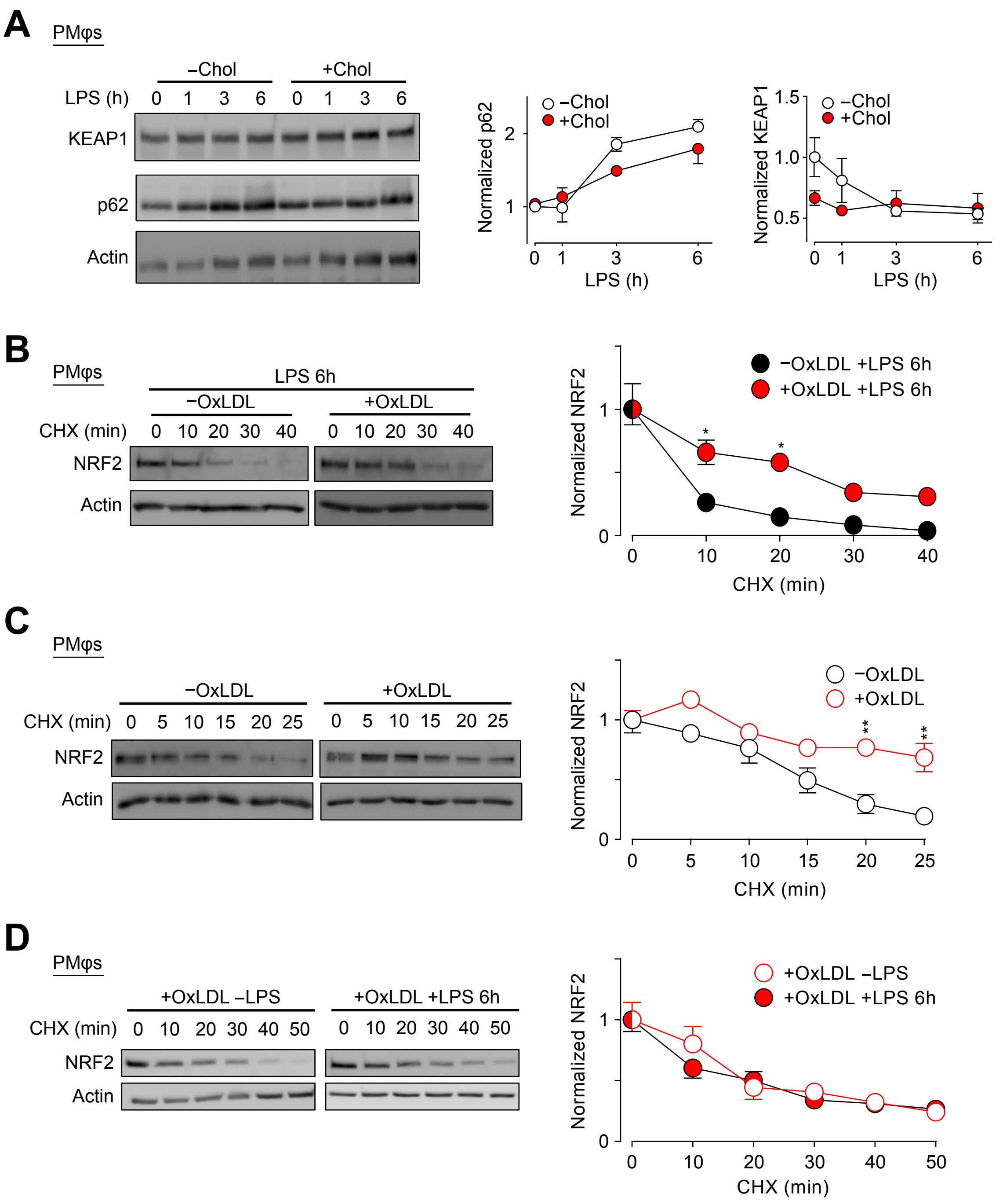

Supplementary Figure 4

(A) Effect of cholesterol (Chol) loading on KEAP1 and p62 protein expression in PMφs stimulated with LPS (0 - 6 h). Representative immunoblots and quantification are shown. Data are normalized to the corresponding actin and the 0 h LPS time point in cells without Chol loading (assigned a value of 1, n = 3). **(B, C)** Effect of oxLDL loading on NRF2 protein stability in PMφs. Representative immunoblots and quantification are shown. **(B)** After LPS stimulation for 6 h, cycloheximide (CHX) was added to block protein synthesis and NRF2 protein abundance was assessed over time. **(C)** LPS stimulation was omitted and CHX was added to cultured PMφs after loading with oxLDL. For both groups (with and without oxLDL loading), data are normalized to the corresponding actin and the pre-CHX time point (assigned a value of 1, n = 3). **(D)** Effect of LPS stimulation on NRF2 protein stability in PMφs loaded with oxLDL. Representative immunoblots and quantification are shown. Cells were first loaded with oxLDL, then stimulated with LPS or control buffer for 6 h prior to the addition of CHX and assessment of NRF2 protein abundance over time. Data were normalized as above (n = 3). The mean ± SEM is plotted in all graphs. Significant differences are determined by a two-way ANOVA with Bonferroni correction (* $P < 0.05$, ** $P < 0.01$).
