# Supplementary figures and images for: Cholesterol accumulation impairs HIF-1α-dependent immunometabolic reprogramming of LPS-stimulated macrophages by upregulating the NRF2 pathway
Source: Sci Rep. 2024 May 15;14:11162. doi: 10.1038/s41598-024-61493-6 (PMC11096387; doi:10.1038/s41598-024-61493-6)

48 kDa —

35 kDa —

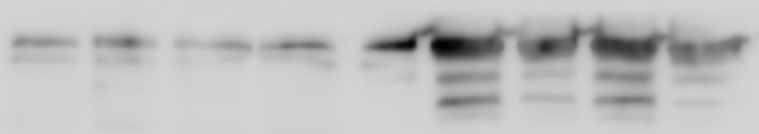

Supplement: Supplementary file 1 — Supplementary Information. [file 41598_2024_61493_MOESM1_ESM.zip › Uncropped blot-Fig1A-Gapdh.pdf]

Figure 1A

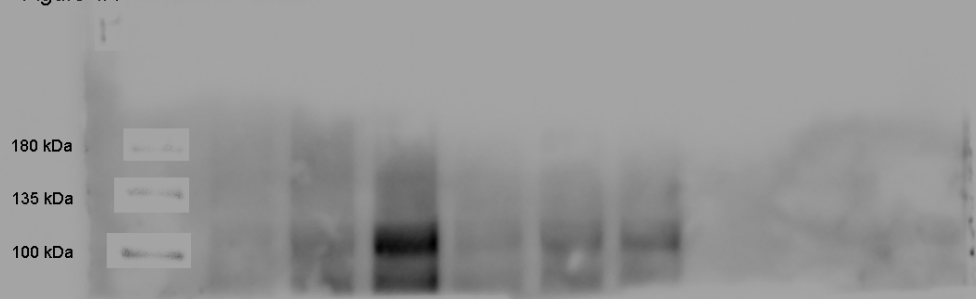

Supplement: Supplementary file 1 — Supplementary Information. [file 41598_2024_61493_MOESM1_ESM.zip › Uncropped blot-Fig1A-HIF1a.pdf]

245 kDa

180 kDa

135 kDa

100 kDa

—

—

—

—

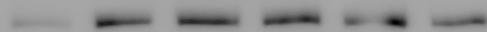

Supplement: Supplementary file 1 — Supplementary Information. [file 41598_2024_61493_MOESM1_ESM.zip › Uncropped blot-Fig1A-HIF2a.pdf]

Figure 1A

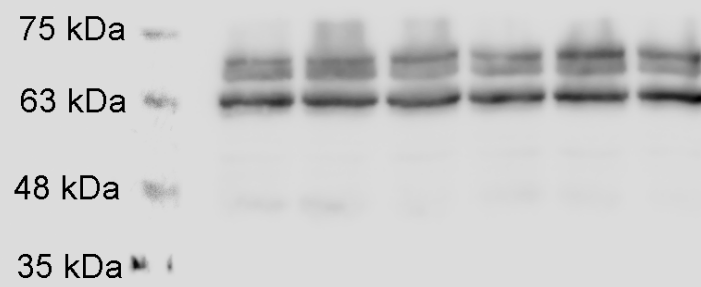

Supplement: Supplementary file 1 — Supplementary Information. [file 41598_2024_61493_MOESM1_ESM.zip › Uncropped blot-Fig1A-LaminAC.pdf]

Figure 1C

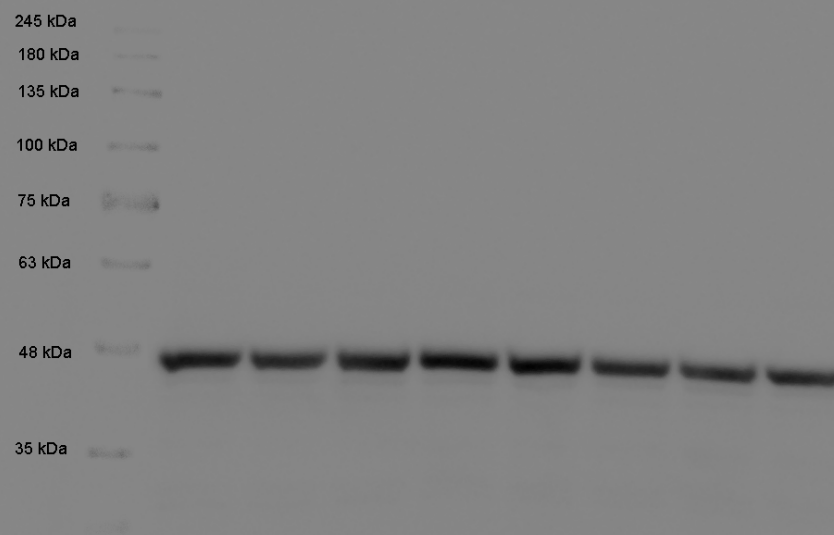

Supplement: Supplementary file 1 — Supplementary Information. [file 41598_2024_61493_MOESM1_ESM.zip › Uncropped blot-Fig1C-actin+Chol.pdf]

Figure 1C

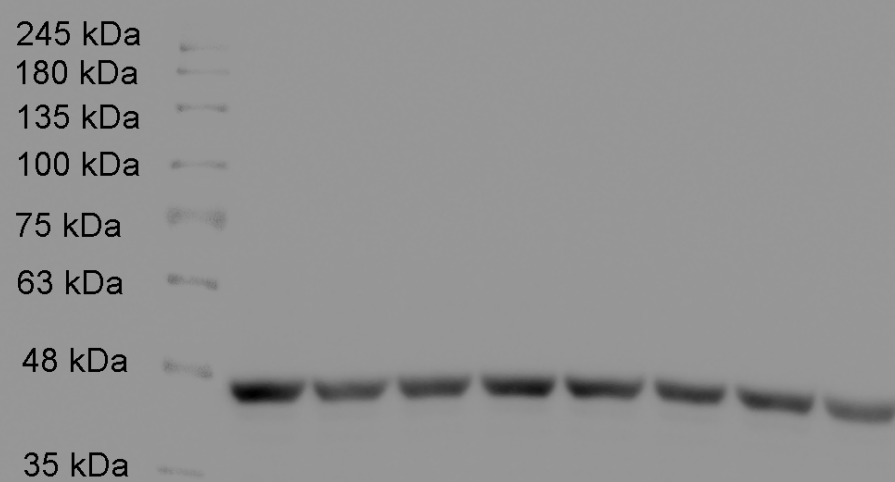

Supplement: Supplementary file 1 — Supplementary Information. [file 41598_2024_61493_MOESM1_ESM.zip › Uncropped blot-Fig1C-actin-Chol.pdf]

Figure 1C

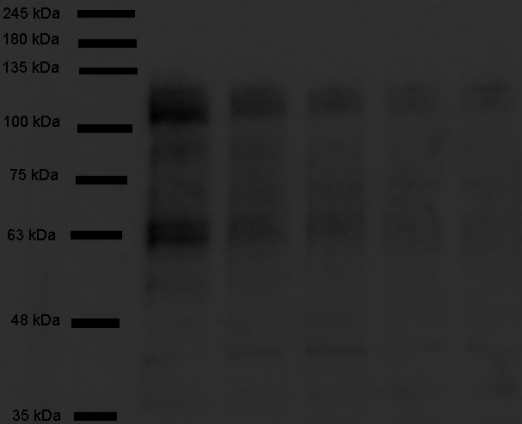

Supplement: Supplementary file 1 — Supplementary Information. [file 41598_2024_61493_MOESM1_ESM.zip › Uncropped blot-Fig1C-HIF1a+Chol.pdf]

Figure 1C

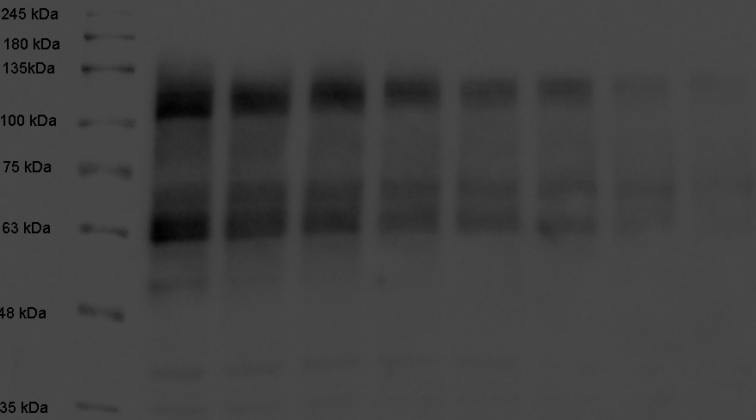

Supplement: Supplementary file 1 — Supplementary Information. [file 41598_2024_61493_MOESM1_ESM.zip › Uncropped blot-Fig1C-HIF1a-Chol.pdf]

Figure 1G

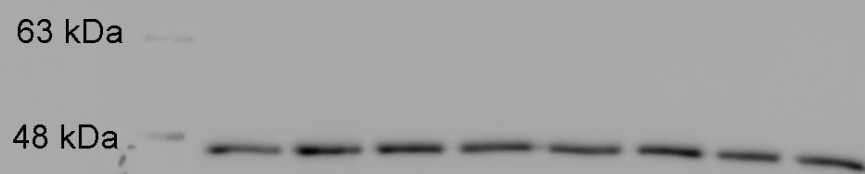

Supplement: Supplementary file 1 — Supplementary Information. [file 41598_2024_61493_MOESM1_ESM.zip › Uncropped blot-Fig1G-actin.pdf]

Figure 1G

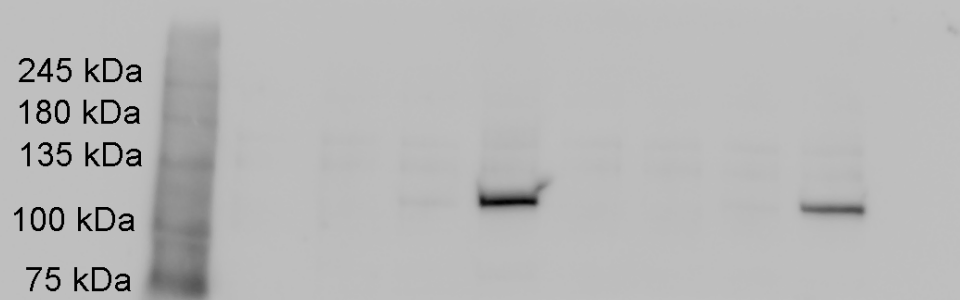

Supplement: Supplementary file 1 — Supplementary Information. [file 41598_2024_61493_MOESM1_ESM.zip › Uncropped blot-Fig1G-Nos2.pdf]

Figure 1G

100 kDa

75 kDa

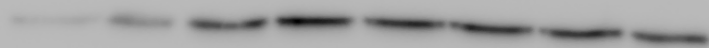

Supplement: Supplementary file 1 — Supplementary Information. [file 41598_2024_61493_MOESM1_ESM.zip › Uncropped blot-Fig1G-Nox2.pdf]

Figure 2A

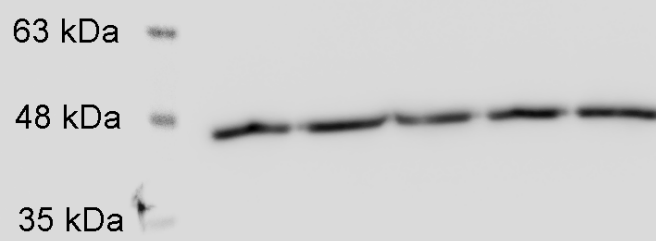

Supplement: Supplementary file 1 — Supplementary Information. [file 41598_2024_61493_MOESM1_ESM.zip › Uncropped blot-Fig2A-actin-Cre+ve+chol.pdf]

63 kDa

48 kDa

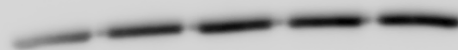

Supplement: Supplementary file 1 — Supplementary Information. [file 41598_2024_61493_MOESM1_ESM.zip › Uncropped blot-Fig2A-actin-Cre+ve-chol.pdf]

Figure 2A

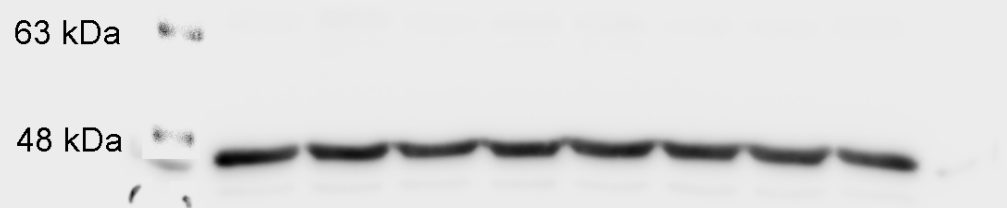

Supplement: Supplementary file 1 — Supplementary Information. [file 41598_2024_61493_MOESM1_ESM.zip › Uncropped blot-Fig2A-actin-Cre-ve+chol.pdf]

Figure 2A

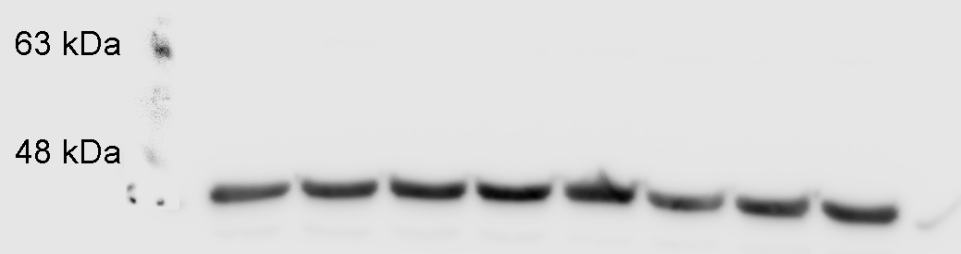

Supplement: Supplementary file 1 — Supplementary Information. [file 41598_2024_61493_MOESM1_ESM.zip › Uncropped blot-Fig2A-actin-Cre-ve-chol.pdf]

Figure 2A

245 kDa

180 kDa

135 kDa

100 kDa

75 kDa

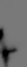

Supplement: Supplementary file 1 — Supplementary Information. [file 41598_2024_61493_MOESM1_ESM.zip › Uncropped blot-Fig2A-HIF1a-Cre+ve+chol.pdf]

Figure 2A

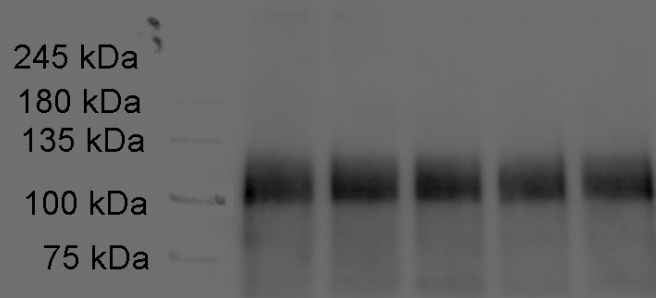

Supplement: Supplementary file 1 — Supplementary Information. [file 41598_2024_61493_MOESM1_ESM.zip › Uncropped blot-Fig2A-HIF1a-Cre+ve-chol.pdf]

Figure 2A

245 kDa

180 kDa

135 kDa

100 kDa

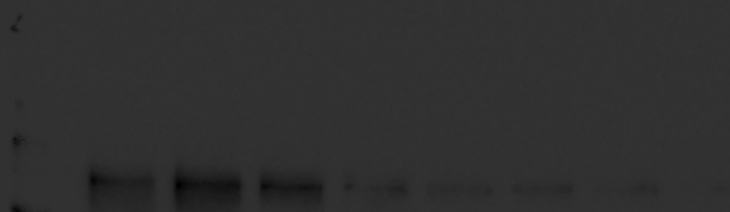

Supplement: Supplementary file 1 — Supplementary Information. [file 41598_2024_61493_MOESM1_ESM.zip › Uncropped blot-Fig2A-HIF1a-Cre-ve+chol.pdf]

Figure 2A

245 kDa

180 kDa

135 kDa

100 kDa

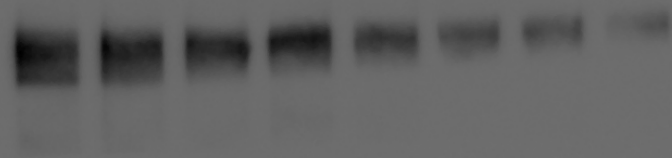

Supplement: Supplementary file 1 — Supplementary Information. [file 41598_2024_61493_MOESM1_ESM.zip › Uncropped blot-Fig2A-HIF1a-Cre-ve-chol.pdf]

Figure 2B

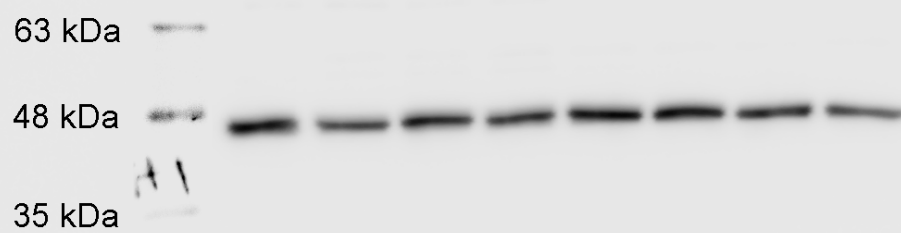

Supplement: Supplementary file 1 — Supplementary Information. [file 41598_2024_61493_MOESM1_ESM.zip › Uncropped blot-Fig2B-actin.pdf]

Figure 2B

245 kDa

180 kDa

135 kDa

100 kDa

75 kDa

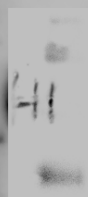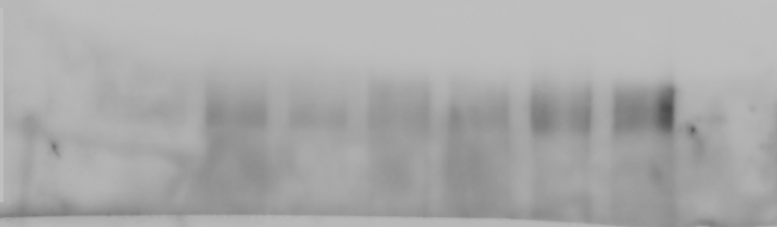

Supplement: Supplementary file 1 — Supplementary Information. [file 41598_2024_61493_MOESM1_ESM.zip › Uncropped blot-Fig2B-HIF1a.pdf]

Figure 2E

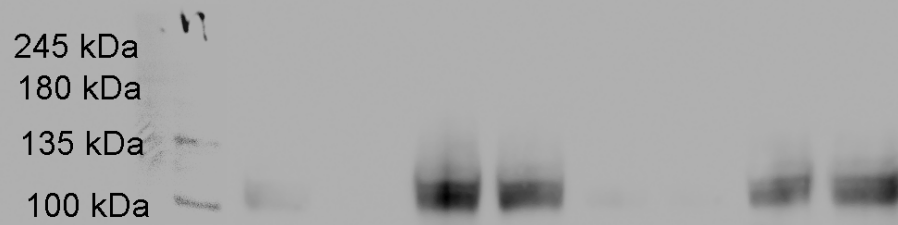

Supplement: Supplementary file 1 — Supplementary Information. [file 41598_2024_61493_MOESM1_ESM.zip › Uncropped blot-Fig2E-HIF1a.pdf]

Figure 2E

245 kDa

135 kDa

100 kDa

75 kDa

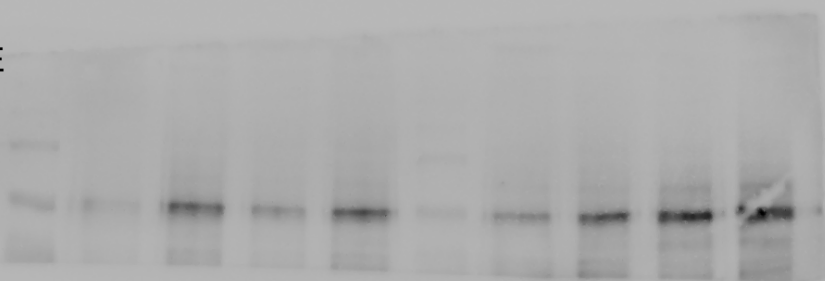

Supplement: Supplementary file 1 — Supplementary Information. [file 41598_2024_61493_MOESM1_ESM.zip › Uncropped blot-Fig2E-HydroxyHIF1a.pdf]

Figure 2E

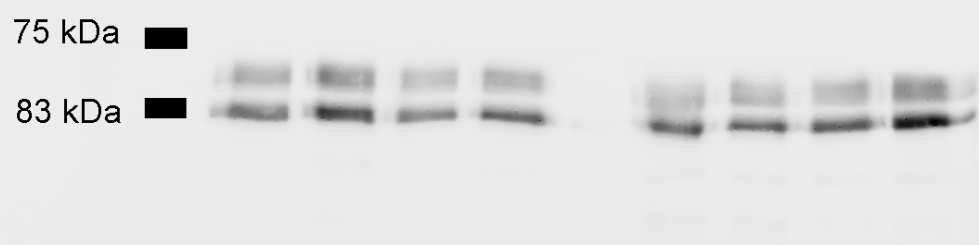

Supplement: Supplementary file 1 — Supplementary Information. [file 41598_2024_61493_MOESM1_ESM.zip › Uncropped blot-Fig2E-LaminAC.pdf]

Figure 3A

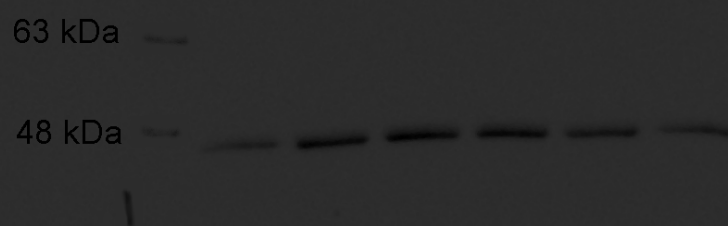

Supplement: Supplementary file 1 — Supplementary Information. [file 41598_2024_61493_MOESM1_ESM.zip › Uncropped blot-Fig3A-actin.pdf]

Figure 3A

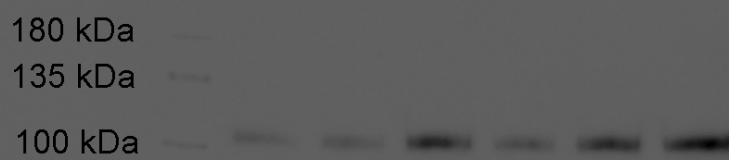

Supplement: Supplementary file 1 — Supplementary Information. [file 41598_2024_61493_MOESM1_ESM.zip › Uncropped blot-Fig3A-Nrf2.pdf]

Figure 3C

48 kDa 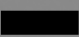

35 kDa 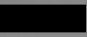

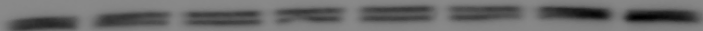

Supplement: Supplementary file 1 — Supplementary Information. [file 41598_2024_61493_MOESM1_ESM.zip › Uncropped blot-Fig3C-Nqo1.pdf]

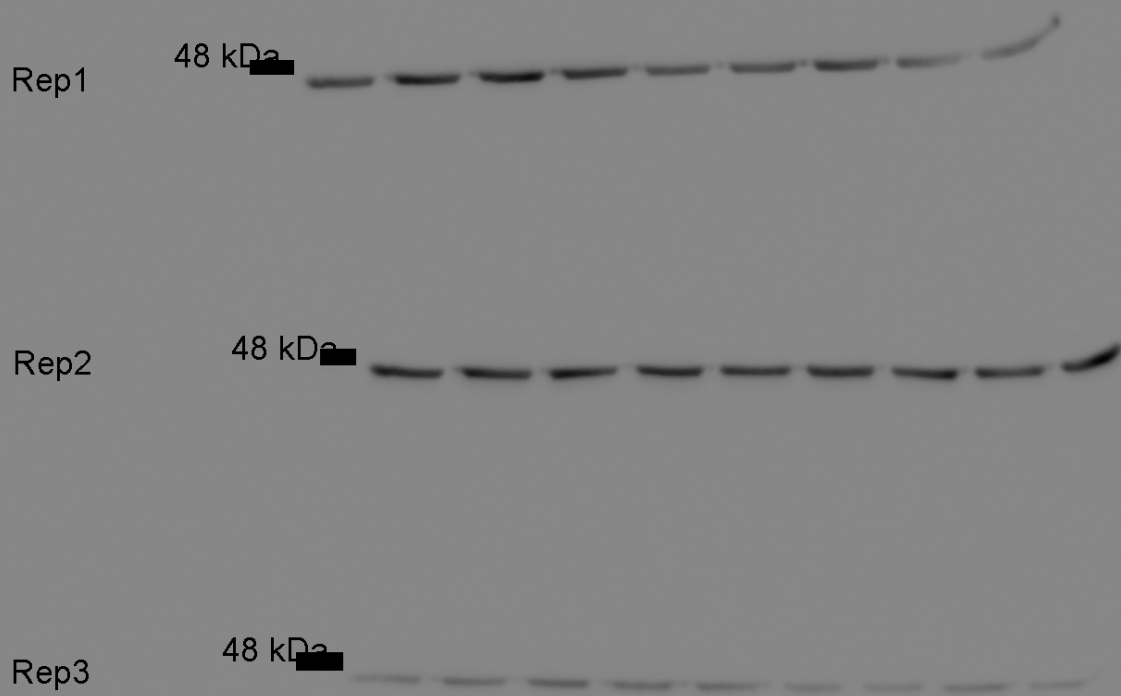

Supplement: Supplementary file 1 — Supplementary Information. [file 41598_2024_61493_MOESM1_ESM.zip › Uncropped blot-Fig3E-actin.pdf]

Figure 3E

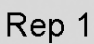

63 kDa

48 kDa

Rep 2

63 kDa

48 kDa

63 kDa

Rep 3

48 kDa

Supplement: Supplementary file 1 — Supplementary Information. [file 41598_2024_61493_MOESM1_ESM.zip › Uncropped blot-Fig3E-Keap1.pdf]

Rep1

100 kDa ■

75 kDa ■

Rep2

100 kDa ■

75 kDa ■

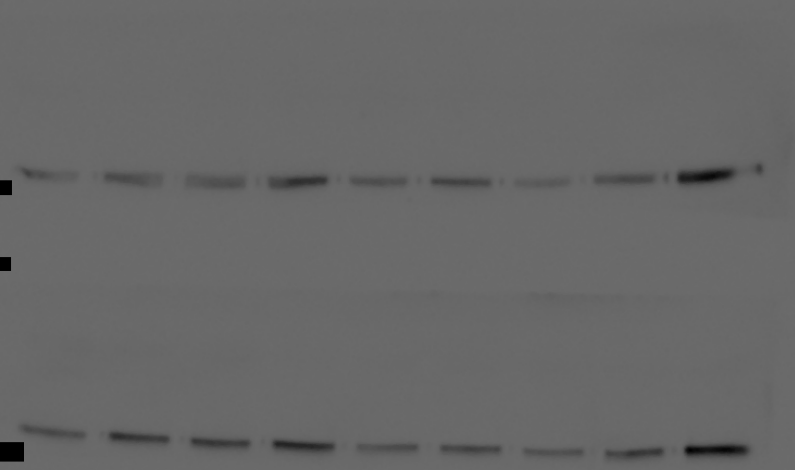

Supplement: Supplementary file 1 — Supplementary Information. [file 41598_2024_61493_MOESM1_ESM.zip › Uncropped blot-Fig3E-Nrf2.pdf]

Figure 4B

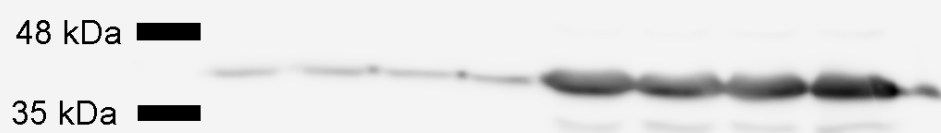

Supplement: Supplementary file 1 — Supplementary Information. [file 41598_2024_61493_MOESM1_ESM.zip › Uncropped blot-Fig4B-gapdh.pdf]

Figure 4B

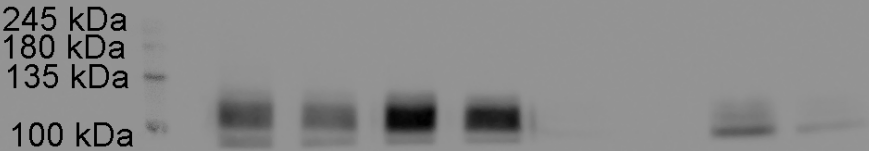

Supplement: Supplementary file 1 — Supplementary Information. [file 41598_2024_61493_MOESM1_ESM.zip › Uncropped blot-Fig4B-HIF1a.pdf]

Figure 4B

75 kDa ■

63 kDa ■

48 kDa ■

35 kDa ■

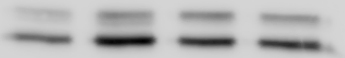

Supplement: Supplementary file 1 — Supplementary Information. [file 41598_2024_61493_MOESM1_ESM.zip › Uncropped blot-Fig4B-lamin.pdf]

Figure 5A

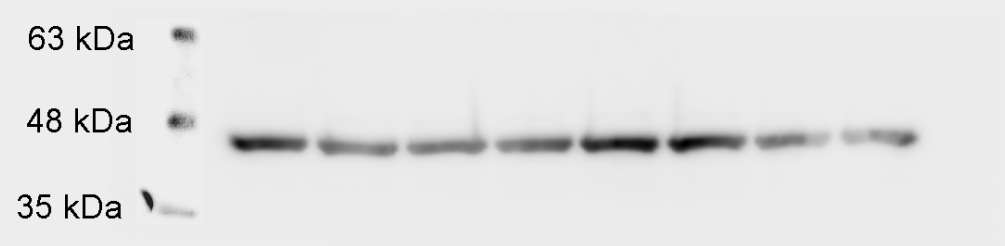

Supplement: Supplementary file 1 — Supplementary Information. [file 41598_2024_61493_MOESM1_ESM.zip › Uncropped blot-Fig5A-actin+chol.pdf]

Figure 5A

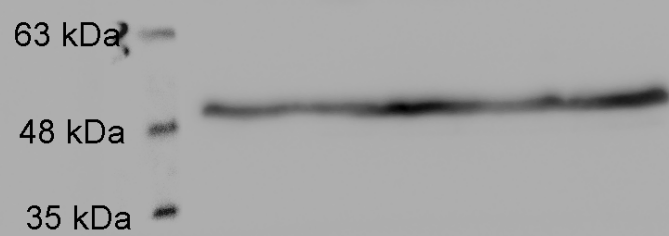

Supplement: Supplementary file 1 — Supplementary Information. [file 41598_2024_61493_MOESM1_ESM.zip › Uncropped blot-Fig5A-actin-chol.pdf]

Figure 5A

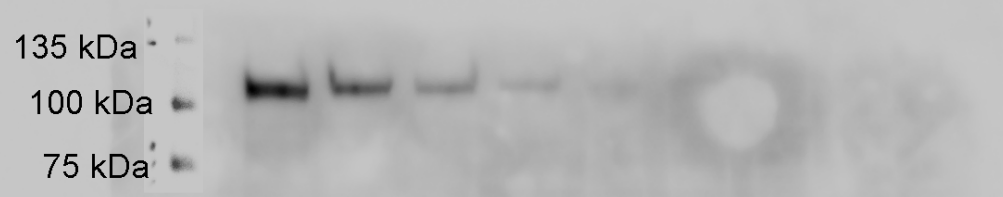

Supplement: Supplementary file 1 — Supplementary Information. [file 41598_2024_61493_MOESM1_ESM.zip › Uncropped blot-Fig5A-Nrf2+chol.pdf]

Figure 5

180 kDa  
135 kDa  
100 kDa  
75 kDa

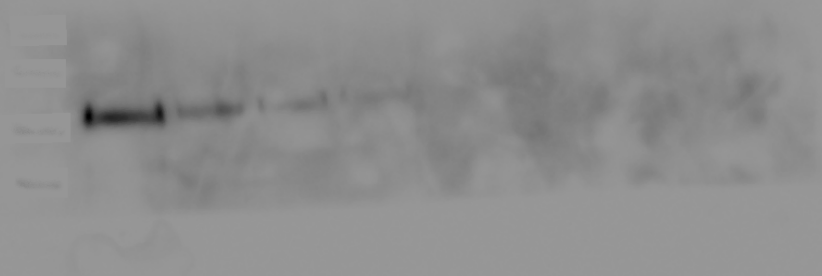

Supplement: Supplementary file 1 — Supplementary Information. [file 41598_2024_61493_MOESM1_ESM.zip › Uncropped blot-Fig5A-Nrf2-chol.pdf]

Figure 4B

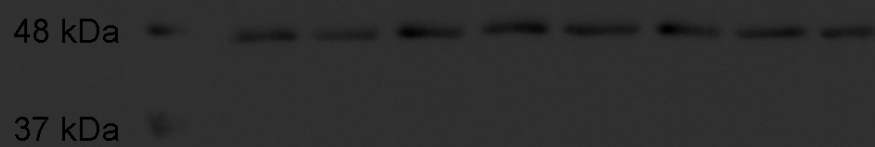

Supplement: Supplementary file 1 — Supplementary Information. [file 41598_2024_61493_MOESM1_ESM.zip › Uncropped blot-Fig5B-actin+chol.pdf]

Figure 5B

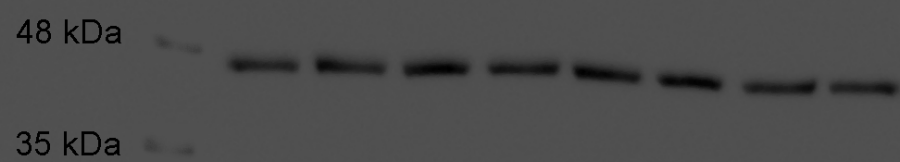

Supplement: Supplementary file 1 — Supplementary Information. [file 41598_2024_61493_MOESM1_ESM.zip › Uncropped blot-Fig5B-actin-chol.pdf]

Figure 5B

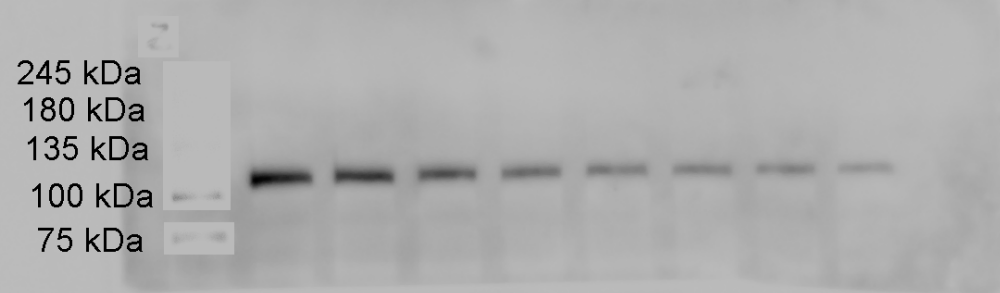

Supplement: Supplementary file 1 — Supplementary Information. [file 41598_2024_61493_MOESM1_ESM.zip › Uncropped blot-Fig5B-Nrf2+chol.pdf]

Figure 5B

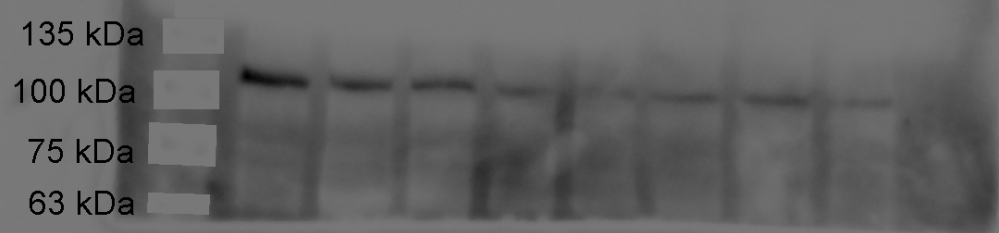

Supplement: Supplementary file 1 — Supplementary Information. [file 41598_2024_61493_MOESM1_ESM.zip › Uncropped blot-Fig5B-Nrf2-chol.pdf]

Figure 5C

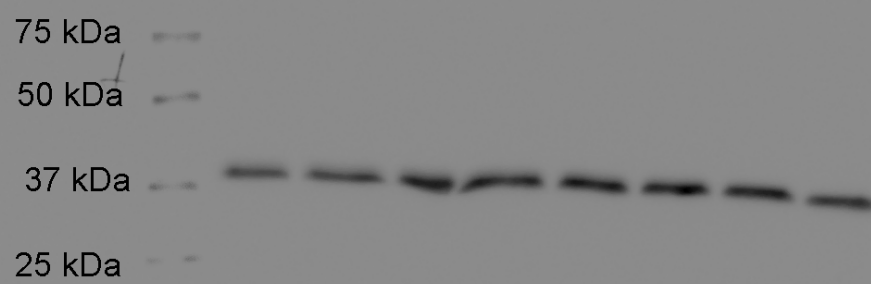

Supplement: Supplementary file 1 — Supplementary Information. [file 41598_2024_61493_MOESM1_ESM.zip › Uncropped blot-Fig5C-actin+LPS.pdf]

Figure 5C

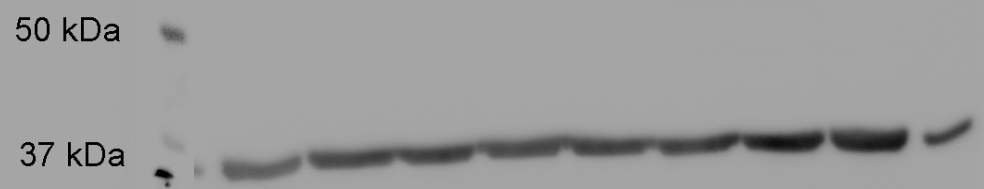

Supplement: Supplementary file 1 — Supplementary Information. [file 41598_2024_61493_MOESM1_ESM.zip › Uncropped blot-Fig5C-actin-LPS.pdf]

Figure 5C

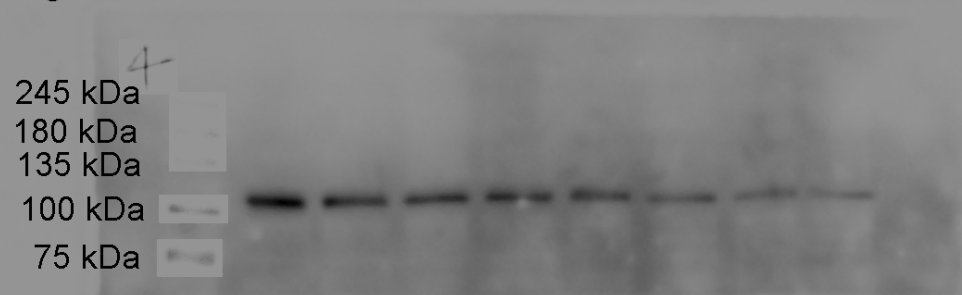

Supplement: Supplementary file 1 — Supplementary Information. [file 41598_2024_61493_MOESM1_ESM.zip › Uncropped blot-Fig5C-Nrf2+LPS.pdf]

Figure 5C

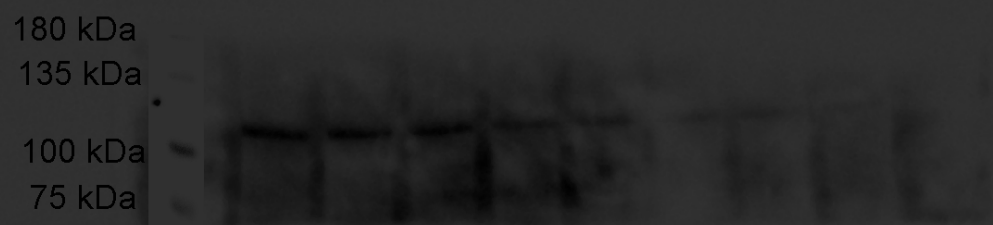

Supplement: Supplementary file 1 — Supplementary Information. [file 41598_2024_61493_MOESM1_ESM.zip › Uncropped blot-Fig5C-Nrf2-LPS.pdf]

Figure 5E

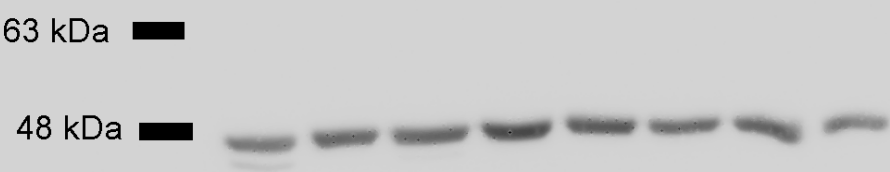

Supplement: Supplementary file 1 — Supplementary Information. [file 41598_2024_61493_MOESM1_ESM.zip › Uncropped blot-Fig5E-actin-Mutant.pdf]

Figure 5E

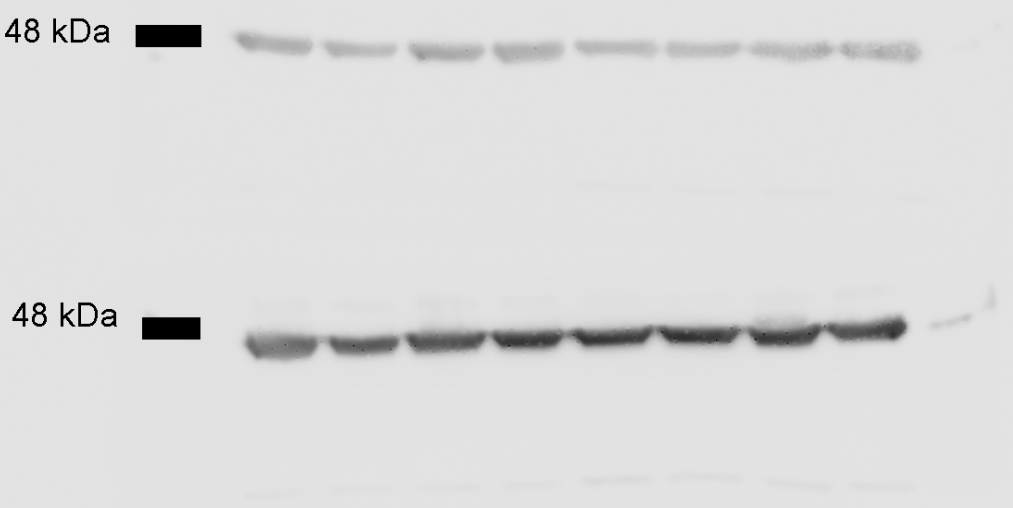

Supplement: Supplementary file 1 — Supplementary Information. [file 41598_2024_61493_MOESM1_ESM.zip › Uncropped blot-Fig5E-actin-WT.pdf]

Figure 5E

100 kDa

63 kDa

48 kDa

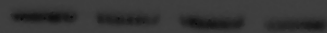

Supplement: Supplementary file 1 — Supplementary Information. [file 41598_2024_61493_MOESM1_ESM.zip › Uncropped blot-Fig5E-mCherry-Mutant.pdf]

Figure 5E

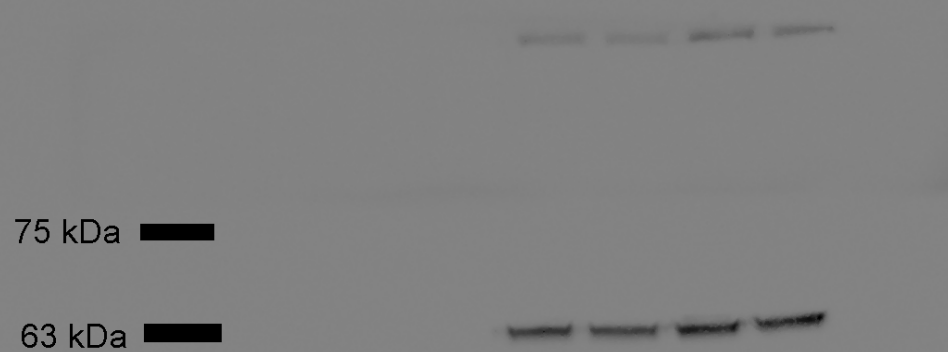

Supplement: Supplementary file 1 — Supplementary Information. [file 41598_2024_61493_MOESM1_ESM.zip › Uncropped blot-Fig5E-mCherry-WT.pdf]

Figure 5E

245 kDa  
180 kDa  
135 kDa  
100 kDa

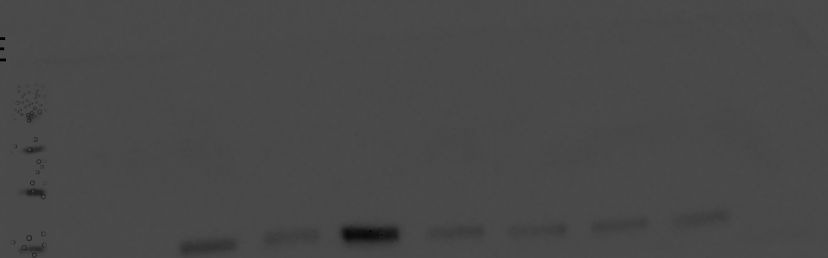

Supplement: Supplementary file 1 — Supplementary Information. [file 41598_2024_61493_MOESM1_ESM.zip › Uncropped blot-Fig5E-Nrf2-Mutant.pdf]

Figure 5E

135 kDa ■

100 kDa ■

75 kDa ■

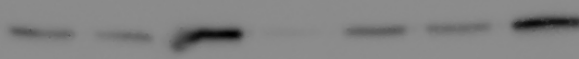

Supplement: Supplementary file 1 — Supplementary Information. [file 41598_2024_61493_MOESM1_ESM.zip › Uncropped blot-Fig5E-Nrf2-WT.pdf]
